# Phenylglyoxal-induced Ana o 3 Modification Reduces Antibody Binding with Minimal Alteration in Protein Structure

**DOI:** 10.1021/acs.jafc.5c10372

**Published:** 2025-09-17

**Authors:** C. Nacaya Brown, Tien Thuy Vuong, Austin T. Weigle, Yu-Jou Chou, Qinchun Rao, Christopher C. Ebmeier, Rebecca A. Dupre, Stephen M. Boue, Brennan Smith, Christopher P. Mattison

**Affiliations:** † Food Processing Sensory Quality, 17123 USDA Agricultural Research Service, New Orleans, Louisiana 70124, United States; ‡ Stored Product Insect and Engineering Research Unit, Center for Grain and Animal Health Research, USDA Agricultural Research Service, Manhattan 66502, United States; § SCINet Program and ARS Center of Excellence, Office of National Programs, USDA Agricultural Research Service, Beltsville, Maryland 20705, United States; ∥ Center for Integrative Nutrition and Food Research, Department of Health, Nutrition, and Food Sciences, 7823 Florida State University, 120 Convocation Way, Tallahassee, Florida 32306, United States; ⊥ Proteomics and Mass Spectrometry Shared Research Resource, Department of Biochemistry, 1877 University of Colorado, Boulder, Colorado 80309, United States

**Keywords:** food allergy, cashew, Ana o 3, 2S albumin, phenylglyoxal, antibody, arginine

## Abstract

The nutritional benefits of nut consumption are complicated by the presence of allergens. Food processing techniques can modify the food protein properties by altering their biophysical characteristics and allergen activity. This study examines the phenylglyoxal-based chemical modification of the cashew nut allergen Ana o 3. Immunoassays with multiple antibodies demonstrate reduced recognition of phenylglyoxal-modified Ana o 3. Mass spectrometry identified multiple Ana o 3 modification sites, including arginine 41, 54, 85, and 111. Circular dichroism, biochemical assays, and molecular simulation indicate that the modifications resulted in minimal protein structure alteration. The research presented here provides insight into Ana o 3 surface chemistry and structure, and it could be applied in the design of alternative forms of immunotherapy to treat cashew nuts and other food allergies.

## Graphical abstract

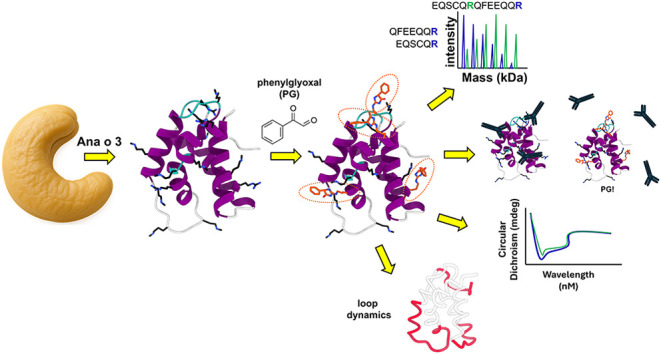

## Introduction

Cashew nuts and other tree nuts have excellent nutritional value and are useful additions to healthy dietary changes. Consumption of cashew nuts has been associated with decreased adolescent and adult obesity markers.^1–4^ Cashew nuts contain beneficial unsaturated fatty acids, including oleic acid, approximately 20% protein, and several micronutrients such as β-carotene, lutein, and phenolic compounds.^5–7^ Characterization of cashew extract subjected to simulated gastrointestinal digestion indicated increased antioxidant peptides and phenolics in the soluble fraction and the potential as a prebiotic fiber source in the insoluble fraction.^8^

Cashew nuts are also a common source of food allergens. Cashew nuts contain at least three allergens, including Ana o 1, Ana o 2, and Ana o 3.^9–12^ Ana o 3 is considered a major cashew nut allergen and is often responsible for life-threatening anaphylactic reactions.^13–15^ Ana o 3, like other 2S albumins, is a water-soluble, heat-stable, and relatively small (∼14 kDa) seed storage protein containing four conserved disulfide bonds made up of two subunits.^14,16^ Immunoglobulin E (IgE) antibodies to Ana o 3 are a useful marker for severe cashew nut allergy,^17–19^ and Ana o 3 retains its structure following heating and lactic acid bacterial fermentation.^20,21^ There are several isoforms of Ana o 3,^12^ but the immunological significance, if any, of these different isoforms is not understood.

Direct or food-processing-based chemical modification can alter the physical properties of food allergens. Modified allergen derivatives with lowered immunogenic activity, termed “allergoids,” have been inspired by research dating back to the early 20th Century.^22^ There are several examples of allergen modification via chemical means to produce allergoids. Potassium cyanate (KCNO) has been used to carbamylate the ε-amino group of lysine residues in grass and ovalbumin allergens, resulting in reduced biological activity.^23^ Peanut allergens have been modified through reduction with a reducing agent and alkylation to reduce allergenic potency.^24–26^ Similarly, application of sodium sulfite and heating were shown to disrupt the structure of the Ana o 3 cashew nut allergen, leading to reduced antibody binding.^27^

Chemical modification schemes could be optimized to develop an arsenal of allergoids whose derivatives represent a hierarchy of allergen immunotherapy agents with varying potency. However, not every chemical modification approach is effective for a given allergen target. For example, Ana o 3 can be chemically modified by Maillard reactions during heating,^20^ but these modifications are low abundance and difficult to identify and characterize.^28,29^ Alternative or preferred modification approaches should take advantage of allergen protein surface physicochemistry and should be applied in a selective manner.

Ana o 3.0101 is predicted to contain 15 arginine residues in the mature protein,^11,12^ which can be leveraged as useful handles for targeted modification. Phenylglyoxal (PG - C_6_H_5_C­(O)­COH) is an organic compound containing both aldehyde and ketone functional groups that can selectively modify arginine residues in proteins.^30,31^ PG is not like other dicarbonyl reagents, including methylglyoxal or glyoxal, which are known to generate a wide range of advanced glycation end products (AGEs) through Maillard-type reactions. PG is a targeted chemical modifier that reacts specifically with the guanidino group of arginine residues in proteins and forms stable cyclic adducts that can be used in biochemical studies to probe arginine function. PG has been used to attach drugs and probes to peptides and proteins,^32^ used in assays to assess citrullinated fibrinogen in arthritis and cancer patients,^33^ and can function as an in vivo probe for RNA structure in yeast.^34^

Here, PG modification of purified Ana o 3 is evaluated by PAGE and immunoassay with a battery of monoclonal anti-Ana o 3 antibodies that are predicted to recognize both linear and conformational epitopes.^35,36^ Mass spectrometry, circular dichroism, and molecular simulation are used to characterize Ana o 3 structural changes as a result of PG treatment. This research improves our understanding of the immunological and structural implications of Ana o 3 modification and may also enable the generation of novel therapeutics to treat food allergy.

## Materials and Methods

### Materials

Ana o 3 was purified as previously described in Mattison et al.^37^ from cashew nuts purchased at a local grocery store. Phenylglyoxal (PG), trinitrobenzenesulfonic acid (TNBSA), and 1-anilinonaphthalene-8-sulfonic acid (ANS) were purchased from Sigma-Aldrich (St. Louis, MO, USA). Precision Plus Protein Dual Color standards (Bio-Rad, Hercules, CA, USA) served as protein molecular mass markers, and Novex 10–20% Tricine gels (Life Technologies, Carlsbad, CA, USA) were used for polyacrylamide gel electrophoresis (PAGE). Ninety-six (96) well maxisorp plates (Thermo Scientific, Waltham, MA, USA) were used for enzyme-linked immunosorbent assay (ELISA). Primary anti-Ana o 3 monoclonal and polyclonal antibodies have been described previously^35^ and were used with infrared dye-labeled secondary antibodies purchased from LI-COR (Lincoln, NE, USA).

### Reaction of Phenylglyoxal with Ana o 3

PG reacts with positively charged arginine residues on proteins.^31^ Purified Ana o 3 (2.75 mg/mL via A280) was diluted 10-fold in 100 mM potassium phosphate buffer (pH 8.0) and incubated with increasing concentrations of PG+ (0.1–10 mM final) for 1 h at room temperature (22 °C) in a 500 μL reaction volume. Following the incubation of at least three independent reactions, samples were placed on ice or frozen before analysis.

### Trinitrobenzenesulfonic Acid (TNBSA) Assay

Ana o 3 primary amine content was evaluated using a 0.01% (v:v) solution of trinitrobenzenesulfonic acid (TNBSA) in a 100 mM sodium bicarbonate buffer (pH 8.5). Triplicate control or PG-modified Ana o 3 samples (500 μL) were incubated for two h at 37 °C in a mini hybridization oven (Bellco Glass Inc., Vineland, NJ, USA) with 250 μL of 0.01% TNBSA reagent. Following incubation, samples were cooled to room temperature, and the absorbance at 420 nm was measured on a DeNovix DS-C spectrophotometer (DeNovix Inc., Wilmington, DE, USA). The average A_420_ was reported for each phenylglyoxal concentration, and the standard deviation between measured points was calculated using eq 1. The percent modification was calculated using eq 2.

1 $$s=\sqrt{\frac{\sum_{i=1}^{N}{\left(x_{i}-\mathrm{x̅}\right)}^{2}}{N-1}}$$

*s* = sample standard deviation

*N* = number of observations

*x*_*i*_ = the average of the observed values of a sample item

*x̅* = the mean value of the observations

2 $$\%\;\mathrm{modification}=\frac{{OD}_{control}-{OD}_{sample}}{{OD}_{control}}\times 100$$

OD_control_ = average absorbance of the control at 420 nm

OD_sample_ = average absorbance of PG-modified Ana o 3 at 420 nm

The average *A*_420_ and percent modification relative to PG concentration were plotted separately, and the standard deviation for each PG concentration is represented by error bars. Analysis of variance (ANOVA, *p* ≤ 0.01), followed by Tukey’s honestly significant difference (HSD, *p* ≤ 0.01), was used to determine statistically significant differences in amine content.

### Polyacrylamide Gel Electrophoresis (PAGE)

Polyacrylamide gel electrophoresis (PAGE) was carried out essentially as described in Mattison et al.^20^ Briefly, 4× NuPAGE sample buffer (Life Technologies, Carlsbad, CA, USA) was added to a final 1× concentration to protein samples (5 μg) with or without 5 mM dithiothreitol (DTT) to disrupt Ana o 3 protein structure and heated at 65 °C for 15 min. Samples were allowed to cool and electrophoresed on a Novex Mini Cell gel rig (Life Technologies, Carlsbad, CA, USA) along with prestained Precision Plus molecular weight markers (Bio-Rad). Proteins were visualized by staining with SimplyBlue SafeStain (Invitrogen, Grand Island, NY, USA) according to the manufacturer’s instructions and scanning at 680 nM with an Odyssey CLx infrared imaging system (LI-COR, Lincoln, NE, USA).

### Immunoblot and Immunoassay

Immunoblotting and immunoassay were carried out essentially as described in Mattison et al.^20^ The 2H5, 5B7F8, 6B9C1, and 19C9A2 anti-Ana o 3 monoclonal antibodies and rabbit anti-Ana o 3 polyclonal sera have been previously described in Mattison et al.^35^ Electrophoresed native Ana o 3 or 10 mM PG-treated Ana o 3 (5 μg) samples (pretreated with loading buffer containing 5 mM DTT to disrupt Ana o 3 structure or loading buffer neat) were transferred to PVDF membrane for immunoblot using iBlot Gel Transfer Stacks (Invitrogen, Grand Island, NY, USA) on an iBlot apparatus for 7 min according to the manufacturer’s instructions. Membranes were blocked with 1% bovine serum albumin (BSA) in phosphate-buffered saline with 0.1% Tween-20 (PBST, pH 7.4). BSA-blocked blots were first incubated with primary antibody (diluted 1:1,000 in PBST), followed by incubation with appropriate IRDye-labeled secondary antibody (LI-COR) for 1 h at room temperature with three 10 mL PBST washes for 5 min in between incubations.

Ninety-six (96) well microtiter plates were coated with 5 μg of control or 10 mM PG-treated Ana o 3 per well in coating buffer (15 mM Na2CO3, 35 mM NaHCO3, pH 9.6) for immunoassay and incubated overnight at 4 °C. After sample removal, 1% BSA in PBST was used to block wells for 1 h at room temperature. After removal of blocking solution, primary (diluted 1:1,000 in PBST) and appropriate secondary antibodies (diluted 1:10,000 in PBST) were sequentially added to wells for 1 h at 37 °C with three 100 μL PBST washes in between incubations. Antibody binding was visualized and quantified by scanning on an Odyssey CLx infrared imaging system (LI-COR). At least three replicates for each sample were used to generate averages, and data were plotted with standard deviations as ± error bars.

### Liquid Chromatography–Mass Spectrometry (LC–MS) Orbitrap Mass Spectrometry

Identification and characterization of PG-modified Ana o 3 residues by MS was carried out using samples diluted in 50 mM Tris–HCl, pH 8.5, as described in Brown et al. (2025) and Hughes et al.^38,39^ Triplicate control and PG-treated Ana o 3 (1 μg) samples were sequentially treated with 5% (w/v) sodium dodecyl sulfate (SDS), 10 mM tris­(2-carboxyethyl)­phosphine (TCEP), and 40 mM chloroacetamide and boiled for 10 min at 95 °C to denature the protein. Carboxylate-functionalized beads (GE Life Sciences, Marlborough, MA, USA) were used to isolate proteins from salts and other impurities, and proteins were digested overnight with Lys-C/Trypsin (Promega, Madison, WI, USA). Digested peptides were removed from beads using a magnetic rack, desalted with an Oasis HLB cartridge (Waters Corp., Milford, MA, USA) according to the manufacturer’s instructions, and dried by vacuum.

Peptidase-digested samples resuspended in water with 3% (v/v) acetonitrile and 0.1% (v/v) trifluoroacetic acid were injected onto a C18 1.7 μm, 130 Å, 75 μm × 250 mm M-class column (Waters Corp., Milford, MA, USA), using a Thermo Ultimate 3000 RSLCnano UPLC. A 2%–20% acetonitrile gradient at 300 nL/min over 100 min eluted peptides into a Q-Exactive HF-X mass spectrometer (Thermo Scientific, Waltham, MA, USA). Precursor mass spectra (MS1) were captured at 120,000 resolution (spanning 380 to 1580 *m*/*z*) with automatic gain control (AGC) 3E6 target and 45 ms maximum injection time. MS2 fragment peptide ion isolation width was 1.4 *m*/*z* with the 12 most intense ions sequenced. MS2 spectra were captured at a resolution of 15,000 and higher energy collision dissociation (HCD) at 30% of normalized collision energy, with a target of 1E5 for AGC and a maximum injection time of 100 ms.

MaxQuant was used to search MS data with peptide tolerance at 20 ppm and fragment mass tolerance at 50 ppm, designating trypsin as the digestion enzyme, and up to two missed cleavages. A custom library containing Ana o 3.0101 (IUIS) and other Ana o 3 isoform sequences (2422.1/UniProt Q8H2B8, 2421.1/UniProt A0A891LTK2/A0A891LW61, and 0638.1/UniProt A0A891LTQ0) described in Reitsma et al.^12^ was used for peptide identification. Cysteine carbamidomethylation (*m*/*z* + 57) was set as a fixed modification and methionine oxidation (*m*/*z* + 16) and phenylglyoxal-arginine (*m*/*z* + 116)^31^ as variable modifications.

### Circular Dichroism

Circular dichroism measurements to assess protein secondary structure were conducted following the method of Tang et al.^40^ All measurements were performed by using a Chirascan V100 CD spectrometer (Applied Photophysics Ltd., Charlotte, NC, USA) at room temperature. Protein samples (1 mg/mL) were warmed to 37 °C before measurement to avoid protein aggregation. A 0.5 cm path length quartz cuvette was used, and each experiment was conducted in triplicate to ensure reproducibility. UV CD spectra were collected across a wavelength range of 200–280 nm, with a step size of 1 nm. The spectral data in the range of 200–250 nm were then analyzed using the BeStSel web server (http://bestsel.elte.hu), applying single-spectrum analysis with a scale factor of 1 to quantify secondary structure elements.

### Particle Size and Charge Analysis

Particle size distribution and ζ-potential of control and PG-modified Ana o 3 samples were characterized using a Malvern Zetasizer Nano Series model ZS90 (Malvern Instruments, Westborough, MA, USA). To analyze particle sizes, samples were repeatedly tested using 173° (back), 90° (side), and 13° (forward) scattering angles. After extensive optimization, consistent detection of the particle sizes was achieved, but only with 13° forward scattering. This angle is specifically suited for detecting even trace amounts of aggregates. Three replicates each of control and PG reacted Ana o 3 samples diluted 1:10 in 10 mM potassium phosphate buffer (pH 7) were placed in a semimicro BrandTech Scientific UV-cuvette, and particle size(s) present in each solution after 30 scans of 10 s each were reported as averages. Following particle size analysis, the same sample solutions were transferred to a Malvern Analytical disposable folded capillary cell to measure the ζ-potential. Each individual replicate was promptly scanned to avoid denaturation during exposure to electric current. Reported values were recorded once count rates were stable within ±10 kcps (kilo counts per second). Charge analysis was reported as the average in millivolts. All measurements were performed in replicates of three, and average values for each measurement type were calculated and plotted against phenylglyoxal concentration. The standard deviation required to create error bars was calculated as shown in eq A above. Analysis of variance (ANOVA), followed by Tukey’s honestly significant difference (HSD), was used to determine statistically significant differences in particle size and charge.

### 1-Anilinonaphthalene-8-Sulfonic Acid (ANS) Binding Assay

Differences in protein folding and conformation between control and PG-treated Ana o 3 samples were evaluated using 1-anilinonaphthalene-8-sulfonic acid (ANS) binding. ANS fluorescence increases when it binds to hydrophobic regions on protein surfaces.^41^ ANS (10 mM stock solution dissolved in DMSO) was added to control and PG-treated Ana o 3 samples to achieve a 10:1 molar ratio of ANS to protein in a total of 1500 μL of distilled water. Samples were incubated in the dark at room temperature for 30 min, and fluorescence measurements were obtained using an Agilent Eclipse fluorescence spectrometer (Santa Clara, CA, USA) immediately following incubation. Sample solutions were transferred to a 10 mm path length quartz cuvette, excited with 380 nm light, and emission was monitored between 400 and 600 nm using 15 scans per sample, a 5 nm slit width, and a +650 mV photo multiplier tube (PMT).

### Protein and Modified Amino Acid Modeling

Ana o 3 and PG-modified Ana o 3 amino acids were modeled using Molecular Operating Environment (MOE, version 2024.0601, Chemical Computing Group, Montreal, QC, Canada).^42^ A model of the Ana o 3.0101 sequence (UniProt Q8H2B8, residues S38-Q135) was generated using the Ber e 1 structure (RCSB PDB ID: 2LVF)^43^ as a template. MOE QuickPrep, without any fixed atoms, was used to prepare all structures by adding missing atoms and small loops, capping termini, connecting disulfide bonds, permitting terminal amides, sulfonamide, and imidazole groups to flip, inserting hydrogens, and resolving ionization states for optimized hydrogen bonding. A PG-modified arginine residue was manually created in MOE with Protein Builder, and a rotamer library for the modified amino acid was generated. Ana o 3 molecules containing single- or quadruple-PG modifications at specific sites (R41, R54, R85, and R111) were created in MOE using Protein Builder. After addition, the PG-modified residues were allowed to repack, and side chains were minimized. MOE “Protein Properties” and “Patch Analyzer” were used to characterize and visualize the predicted changes in protein chemistry, surface area, and structure due to PG-modified residues.

### Molecular Dynamics System Building and Parameterization

PG residues on Ana o 3 MOE models were parametrized using ff19SB protein force fields and the AM1-BCC charge method in antechamber of AMBER24.^44–48^ The OPC water model was selected with the corresponding Li/Merz 12–6 normal usage set for ion parameters.^49–52^ PBRadii was set to mbondi3. The MOE models of Ana o 3 contained residues S38 to Q135. Terminal residues were capped by using the ACE and NME end-cappings. Disulfide bonds among C39 and C87, C52 and C76, C77 and C124, and C89 and C132 were modeled. Ana o 3 model ionization states were modeled at pH 7. Rectangular boxes were constructed with 15 Å solvent buffers against all faces of the protein with 0.15 M NaCl modeled. System construction was completed by using tleap within AmberTools. On average, system topologies consisted of 36,784 total atoms with 8769 water molecules and approximate box dimensions of 74.1 × 72.2 × 65.8 Å^3^.

### Molecular Dynamics Simulations

Classical molecular dynamics simulations were first prepared via minimization, NVT heating, NPT heating, an NPT “hold” with constraints, and a two-step equilibration.^53^ All simulations were performed using AMBER24.^47^ Systems were minimized using 5,000 steps steepest descent and 45,000 steps conjugate gradient, accounting for interactions involving H atoms and without SHAKE. Harmonic restraints of 5.0 kcal mol-1 Å-2 were applied to backbone atoms. NVT heating to 300 K occurred over 3 ns simulations with harmonic restraints applied to all residue atoms using sander.MPI. NPT “hold” simulations maintained a temperature of 300 K using pmemd.MPI. Two 50 ns equilibration runs were performed with pmemd.cuda, first with the same restraints as NVT and NPT heating, and the second without restraints. Production runs were performed for 1000 ns in triplicate for each Ana o 3 system (WT control, R41PG, R54PG, R85PG, R111PG, and quadruple-PG). All simulations were restarted from coordinate files with random velocities; used the Verlet integrator with a 2 fs time step; implemented the SHAKE algorithm for omitting hydrogen interactions;^54^ had a 10 Å Lennard-Jones nonbonded cutoff consistent with AMBER force fields; and used Particle Mesh Ewald for treatment of long-range electrostatics.^55^ Temperature was maintained using a Bussi thermostat with a 2 ps coupling constant.^56^ NPT simulations were equipped with a Monte Carlo barostat for isotropic pressure scaling at a pressure of 1 bar.

### Analysis of Molecular Dynamics Simulations

CPPTRAJ (version 6.24.0) was used to perform root-mean-square fluctuation (RMSF) and root-mean-square deviation (RMSD) calculations over the entirety of each trajectory.^57^ Based on RMSF analyses, CPTPRAJ was used to calculate all phi and psi dihedral angles for residues Q61-C77 and K106 through E112. Exploratory data analysis (EDA) was applied to see which residues’ backbone dihedral angle distributions were most diverse across the different Ana o 3 systems (Supporting Figures 1 and 2). System angle distributions where means were more than 30° different from those of other distributions were considered functionally significant and filtered for clustering. EDA limited data clustering to the following dihedral 21 angles: Q61_ψ_, G63_φ_, R65_φ_, R65_ψ_, Q68_φ_, R69_φ_, Q70_ψ_, E71_ψ_, S72_ψ_, L73_ψ_, G107_φ_, G107_ψ_, E108_φ_, E109_φ_, E109_ψ_, V110_φ_, V110_ψ_, R111_φ_, R111_ψ_, E112_φ_, E112_ψ_.

Dataframes containing angles for each respective simulated system were clustered using self-organizing maps (SOMs) with the Python package MiniSom (version 2.3.5).^58^ SOMs have been demonstrated to be an effective unsupervised learning technique for clustering and visualization for molecular dynamics data across a variety of time scales.^59–61^ Based on previously published heuristics, a grid search was performed across each system’s EDA dihedral dataframe to optimize clustering for representative states.^59–62^ EDA dataframes were mean-normalized and unit variance scaled using the StandardScaler function of scikit-learn (version 1.5.2). SOM neuron count scanned the set of [5, 100], incrementing by five. The neighborhood function was Gaussian and sampled σ values spanning [1.00, 6.25] and incrementing by 0.25. The learning rate was 0.5. The epoch number used was 5000. Hyperparameter combinations were evaluated by minimization of the quantization error and distortion measure within the top 10% of the topographic error-minimized models. The final hyperparameter settings used were: (WT: 65 × 65 neurons, 5.25 σ), (R41PG: 45 × 45 neurons, 5.25 σ), (R54PG: 50 × 50 neurons, 4.75 σ), (R85PG: 60 × 60 neurons, 5.25 σ), (R111PG: 70 × 70 neurons, 5.50 σ), and (quadruple-PG: 40 × 40 neurons, 4.75 σ). Neuronal weights from the best-matching units were clustered via hierarchical agglomerative clustering to assign macrostate labels. In accordance with published protocols, the final cluster count was determined from a reasonable range, from 5 to 20, by taking the second maximum from the silhouette method (Supporting Table 1).^61^

MM/GBSA (version 14.0) calculations implemented within AMBER24 were performed for ranking representative states within Ana o 3 conformational clusters.^63^ To minimize bias in the selection of representative states, poses with the lowest MM/GBSA free energy from SOM-determined clusters were visualized. Cluster-derived poses with the highest root-mean-square deviation (RMSD) for residues E59Cα–E71Cα were also visualized. Cluster-derived stable poses may converge to similar conformational flexibility;^43,64^ therefore, trajectories of clustered frames have been provided as Supporting files. GROMACS 2024 was used to perform DSSP calculations on each trajectory.^65,66^ Trajectories were visualized using VMD (version 1.9.4a57), and protein snapshots were rendered using ChimeraX (version 1.8).^67–70^ Computational analyses were plotted using Matplotlib 3.9.2 and Seaborn 0.13.2. Python 3.9 was used for all of the codes mentioned above.

## Results

### PG Modification Decreases Ana o 3 Amine Content

Purified Ana o 3 was combined with phenylglyoxal (PG) in potassium phosphate buffer to allow arginine residues to form PG derivatives. The TNBSA assay was used to quantitatively evaluate the extent of arginine modification in PG-treated (PG-Ana o 3) and untreated Ana o 3. Samples of Ana o 3 reacted with PG indicated there was a linear decrease in the primary Ana o 3 amine content as the PG concentration increased from 0 to 1 mM (Figure 1A). The 10 mM PG reaction suggested the reaction had reached saturation, as no further decrease in amine content was observed. The TNBSA data were converted to estimate the percentage of primary amines within Ana o 3 modified at each PG concentration. At 1.0 mM PG, approximately 50% of the TNBSA reactive Ana o 3 amine content had been modified by PG, and we observed no further decrease in the 10 mM PG reaction based on the TNBSA assay results (Figure 1B).

**Figure 1:**
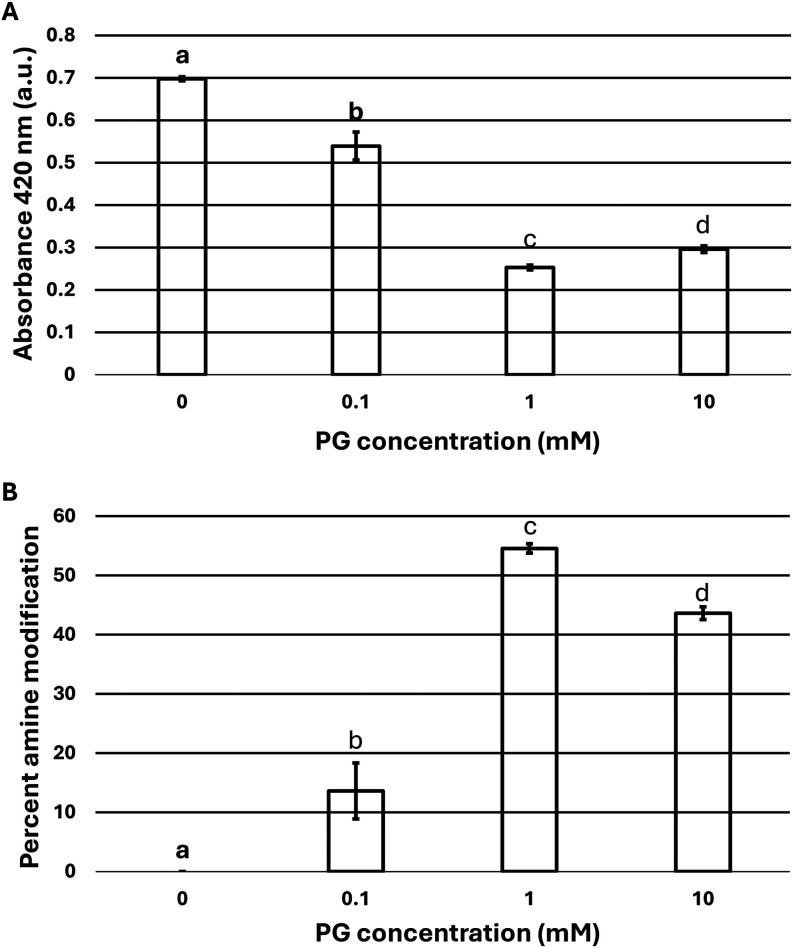
Phenylglyoxal (PG) treated Ana o 3 has reduced amine content. Ana o 3 primary amine content was assessed by the TNBSA assay. The change in the primary amine content of cashew protein Ana o 3 following a 1 h incubation with varied concentrations of PG as reflected in the change of absorbance at 420 nM (A) and expressed as a percentage compared to untreated Ana o 3 (B). PG concentrations (0–10 mM) are indicated on the *x*-axis with absorbance units at 420 nM (A) and the percentage of modified arginine amine content (B) on the *y*-axis. Statistically significant differences in mean values are indicated by letters above the bars in each chart.

### PG Treatment of Ana o 3 Reduces Antibody Recognition

PG-Ana o 3 and control samples were then evaluated by gel and immunoblot in the absence or presence of a pretreatment with dithiothreitol (DTT) as a means to disrupt cystine disulfide bonds and Ana o 3 structure. PAGE of the reaction samples indicated a shift toward less distinct, slower migrating forms of Ana o 3 following PG treatment (Figure 2). The PG-Ana o 3 samples migrated as more diffuse bands, spanning a greater range of molecular masses compared to the control (Figure 2A). Similarly, while the PG-Ana o 3 sample pretreated with DTT allowed visualization of individual subunits, the subunit bands were also more diffuse, although the effect was less apparent (Figure 2B).

**Figure 2:**
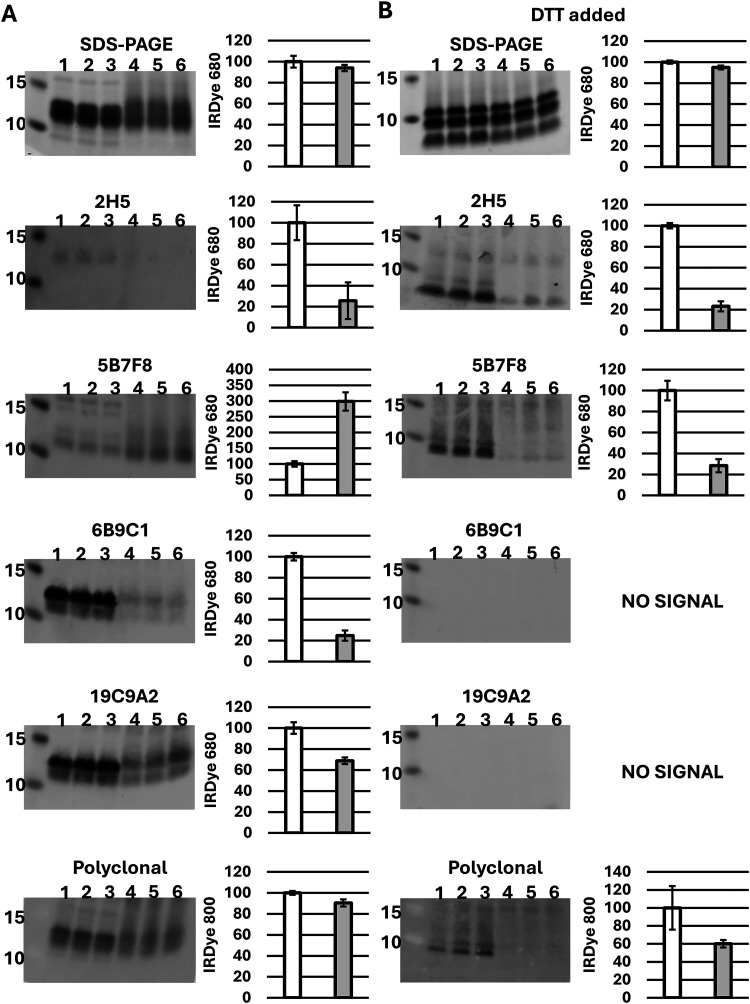
Phenylglyoxal (PG) induced Ana o 3 modifications reduce antibody binding. PAGE and immunoblots of control and 10 mM PG-modified Ana o 3 (A) and those samples additionally pretreated with dithiothreitol (DTT) to disrupt the Ana o 3 structure (B). Immunoblots were individually probed with anti-Ana o 3 monoclonal antibodies 2H5, 5B7F8, 6B9C1, and 19C9A2, and a rabbit polyclonal anti-Ana o 3 sera, as indicated. In each panel, lanes 1–3 are untreated control Ana o 3 samples, and lanes 4–6 are PG-modified Ana o 3. Molecular mass markers are indicated on the left of each blot. Quantified IRDye signal reflects antibody binding as a percentage of the untreated Ana o 3 control sample and is shown in the chart to the right of each blot with control (Ctrl) signal depicted in white bars and PG-treated Ana o 3 (PG) in gray bars.

PG-Ana o 3 modified samples were also evaluated for recognition by a series of anti-Ana o 3 antibodies. In nearly every case, antibody recognition of PG-treated Ana o 3 decreased compared with the untreated control (Figure 2). For example, recognition of PG-Ana o 3 by the 2H5, 6B9C1, and 19C9A2 monoclonal antibodies was clearly lowered, while recognition by the rabbit anti-Ana o 3 polyclonal sera was less affected (Figure 2A). Surprisingly, the 5B7F8 monoclonal antibody signal was slightly elevated in the PG-Ana o 3 sample compared with the control (Figure 2A). In contrast, antibody recognition for the 2H5, 5B7F8, and the rabbit anti-Ana o 3 polyclonal sera was lowered in PG-Ana o 3-treated samples whose structure was disrupted by DTT (Figure 2B). The 6B9C1 and 19C9A2 monoclonal antibodies had no signal in DTT-pretreated samples because they recognize conformational epitopes dependent upon the proper folding of the protein.

ELISA was used to further evaluate antibody recognition of PG-Ana o 3 compared to the untreated control. In each case, antibody recognition of PG-Ana o 3 was reduced compared to the control untreated Ana o 3 sample (Figure 3). For example, recognition of PG-Ana o 3 by the 2H5 monoclonal antibody was reduced by approximately 30% compared to the control sample, either with or without the loss of Ana o 3 structure after DTT treatment (Figure 3). Similarly, there was a reduction in PG-Ana o 3 recognition by the 5B7F8 monoclonal antibody and the rabbit polyclonal anti-Ana o 3 sera, although the effect was less severe. A more drastic reduction in binding to PG-Ana o 3 was observed with both the 6B9C1 and 19C9A2 antibodies, but these antibodies recognize conformational epitopes, and no binding signal was detected after samples were exposed to DTT to disrupt the Ana o 3 protein structure (Figure 3).

**Figure 3:**
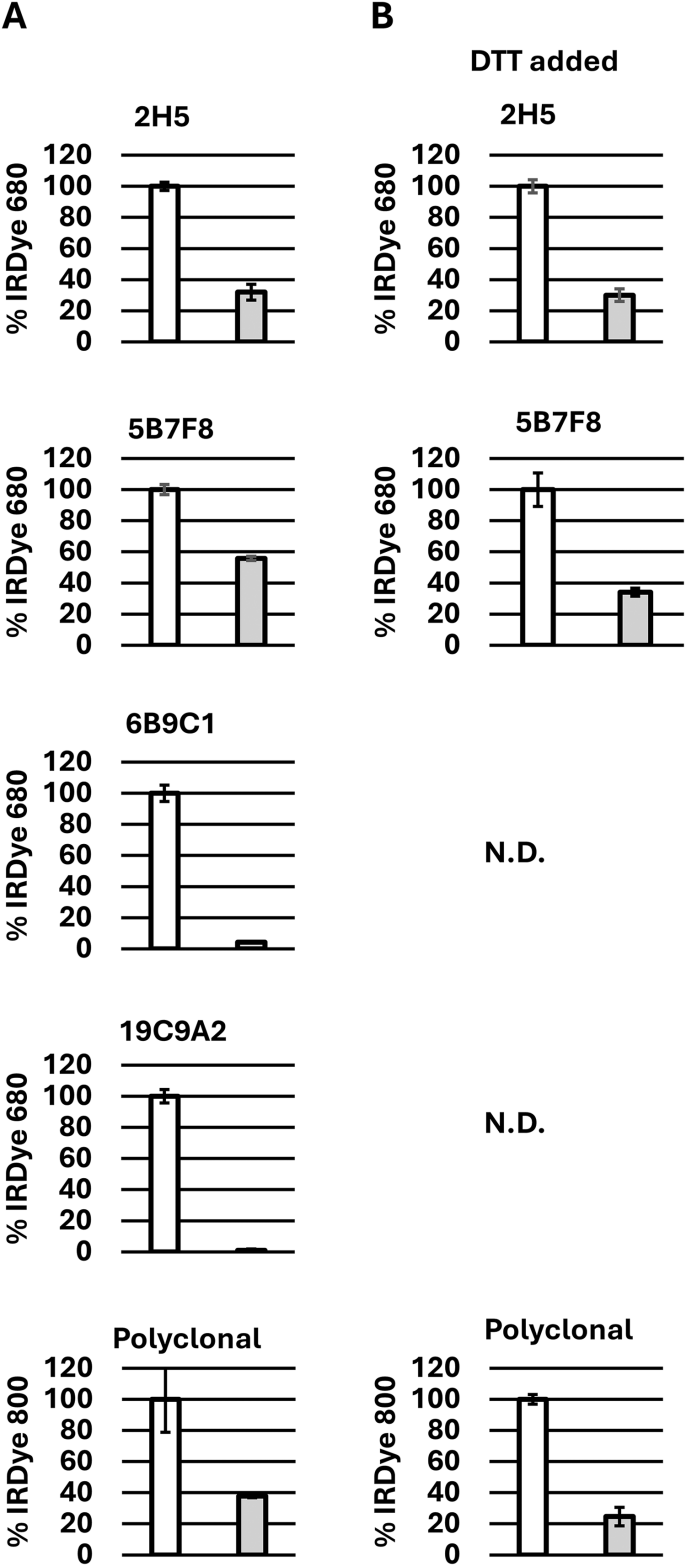
PG modification disrupts anti-Ana o 3 antibody recognition. ELISA comparison of 10 mM PG-Ana o 3 (gray) and untreated Ana o 3 control (white) binding by 2H5, 5B7F8, 6B9C1, and 19C9A2 monoclonal antibodies and a rabbit polyclonal anti-Ana o 3 sera. The *y*-axis represents the percentage of antibody binding to the untreated control sample. Samples were tested without (A) and after exposure to DTT (B) to disrupt the Ana o 3 structure. Bars represent the average of at least three replicates, and standard deviations are indicated as error bars. “N.D”. indicates that no signal was detected.

### Mass Spectrometric Identification of PG-Modified Ana o 3 Arginine Residues

The collection of anti-Ana o 3 antibodies used here is predicted to recognize different epitopes on Ana o 3, and in each case, binding was decreased in the PG-Ana o 3 samples. This observation, along with the TNBSA assay results, suggests multiple Ana o 3 arginine residues react with PG. Indeed, MS analysis of control and PG-modified Ana o 3 indicated numerous arginine residues were chemically modified by the PG treatment.

Among the Ana o 3 peptides observed, several arginine residues were modified, but within each of the three Ana o 3 isoforms (2422.1/UniProt Q8H2B8, 2421.1/UniProt A0A891LTK2/A0A891LW61, and 0638.1/UniProt A0A891LTQ0), there was selectivity in which arginine residues were modified (Figure 4 and Supporting Data File). In addition, among the PG-modified arginine residues, there were four in the 2422.1/UniProt Q8H2B8 Ana o 3 isoform that were routinely observed at relative intensities greater than those of the others. These included the R41, R54, R85, and R111 modification sites (Figure 4). MS/MS fragmentation was used to confirm the identity of these modified sites within Ana 3 (Supporting Figure 3). The preference for modification at these sites likely reflects the abundance of that isoform within the purified Ana o 3 preparation and/or the reactivity of specific arginine residues with PG within the isoform.

**Figure 4:**
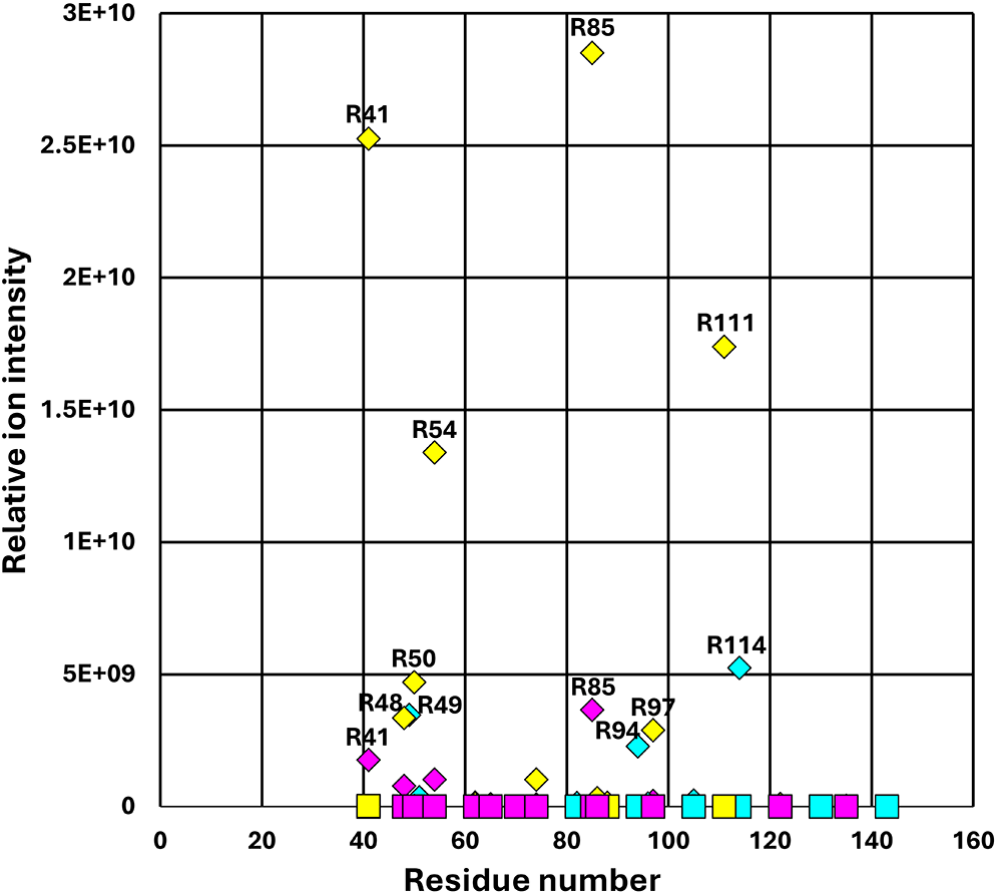
Mass spectrometric (MS) identification of PG-induced Ana 3 modification. Control (squares) and 10 mM PG-Ana o 3 (diamonds) samples were trypsin-treated and evaluated by MS to identify specific PG modifications resulting in a + 116 amu change to peptide mass. Each of the three Ana o 3 isoforms (2422.1/UniProt Q8H2B8 in yellow, 2421.1/UniProt A0A891LTK2/A0A891LW61 in magenta, and 0638.1/UniProt A0A891LTQ0A in turquoise) is shown, and a representative plot of independent triplicate samples is shown. Relative ion intensity is indicated on the *y*-axis, and residue number is indicated on the *x*-axis.

### PG Treatment Slightly Alters Ana o 3 Secondary Structure

The immunoblot and MS data indicated that PG-induced arginine modifications at several sites in Ana 3 were reducing antibody binding. The changes in antibody recognition could result from modification of an arginine directly within or adjacent to an antibody epitope, a structural change in the Ana o 3 conformation, or both. Circular dichroism was used to evaluate Ana o 3 structure prior to and following treatment with PG. The control and PG-modified Ana o 3 spectra displayed characteristic minima around 208 and 222 nm,^71^ indicative of α-helical content (Figure 5).

**Figure 5:**
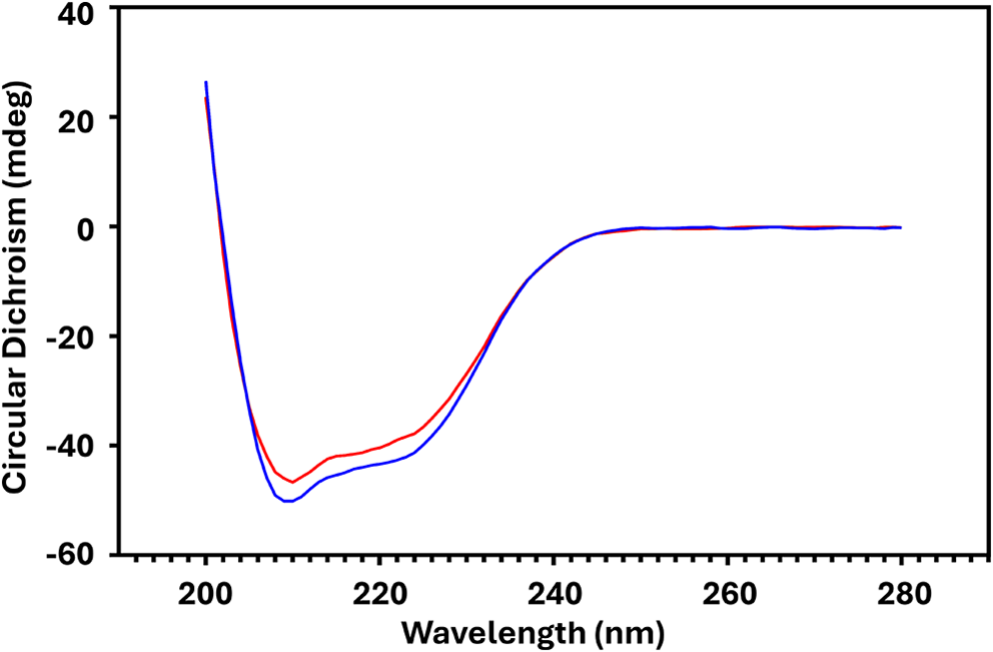
PG-induced arginine modification slightly alters Ana o 3 structure. Circular dichroism analysis of Ana o 3 control (blue) and 10 mM PG-treated Ana o 3 (red), spanning wavelengths from 200 to 280 nm (*x*-axis). Raw molar ellipticity in millidegrees (mdeg) is indicated on the *y*-axis and wavelength on the *x*-axis.

The control sample showed a slightly more pronounced minimum at 222 nm, suggesting a slightly higher α-helical content compared to that of the PG-treated sample. This was consistent with a decrease in regular α-helix structure from 19% in the control to 13.4% in the PG-treated sample, as shown in Table 1. The similarity in the overall shape of the curves implied that, despite some very slight structural alterations from the PG treatment, including the minor reduction in regular helices and a slight increase in disordered regions, the fundamental types of secondary structures remained largely preserved between the two samples.

**Table 1:** Comparative Analysis of Secondary Structure Components in Control and Phenylglyoxal (PG)-Treated Ana o 3

secondary structure component	control (%)	PG (%)	percentage difference
**Helix (%)**	**32.7**	**26.4**	–**19.3**
*regular*	19.0	13.4	–29.5
*distorted*	13.7	13.0	–5.1
**Antiparallel (%)**	**14.7**	**15.9**	**8.2**
*left-twisted*	0.0	0.3	N/A
*relaxed*	9.3	11.1	19.4
*right-twisted*	5.3	4.5	–15.1
parallel (%)	**6.2**	**7.7**	**24.2**
turn (%)	**9.9**	**9.8**	–**1.0**
others (%)	**36.5**	**40.2**	**10.1**

Table 1 further details these slight structural changes, indicating that while the distorted α-helix content remained relatively stable with a minor decrease from 13.7% to 13%, regular helices were reduced. The PG treatment also induced slight increases in left-twisted (Anti1) and relaxed (Anti2) antiparallel β-sheet structures, with Anti1 rising from 0% to 0.3% and Anti2 from 9.3% to 11.1%. Conversely, the right-twisted antiparallel β-sheet structure (Anti3) showed a reduction from 5.3% to 4.5%. Parallel β-sheets experienced an increase from 6.2% to 7.7%, suggesting that these structures may be somewhat stabilized by PG. The turn structure remains largely unaffected, with a negligible change from 9.9% to 9.8%, while the proportion of disordered or random coil regions rises from 36.5% to 40.2%, indicating that PG treatment induced a partial structural disorder or a reduction in organized secondary structures. Overall, these findings suggested that PG treatment led to a slight decrease in regular helical content and an increase in disordered regions and certain β-sheet structures.

### PG Treatment Increases Ana o 3 Particle Size and Decreases Net Protein Charge

Arginine is a positively charged amino acid, and the arginine residues in Ana no. 3 contribute to the overall net charge of the protein. The PG modifications observed here are likely to contribute to differences in charge and particle size of PG-Ana o 3 compared to the untreated control. Control and PG-treated Ana o 3 samples indicated there were changes in the average particle size of PG-modified Ana o 3 relative to the control (Figure 6A).

**Figure 6:**
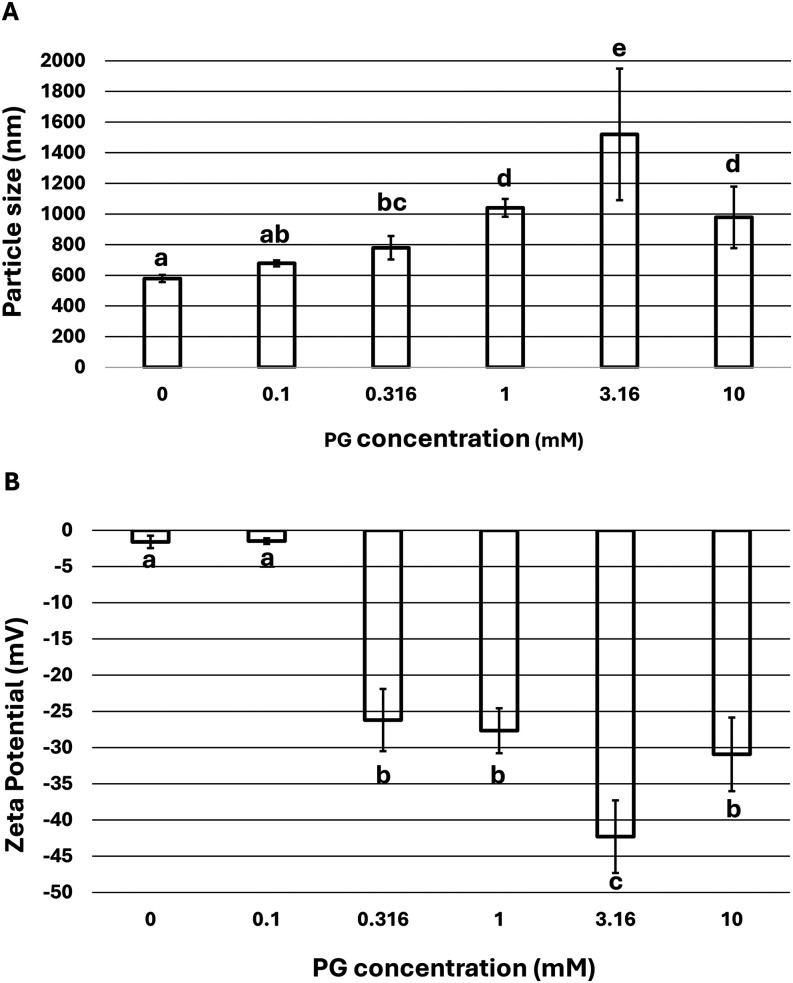
PG treatment alters Ana o 3 particle size and charge. Particle size (A) plotted as average diameter in nanometers and ζ-potential (B) as average charge in millivolts. Bars represent the average of individual triplicate measurements with the indicated PG concentrations on the *x*-axis, and error bars represent the average standard deviation between measurements. Statistically significant differences in sample means are indicated by letters above the bars in the charts.

At low PG concentrations (0.1 mM), there was only a slight increase in average PG-Ana 3 particle size compared to the control (Figure 6A). As the PG concentration increased from 0.316 to 10 mM, the average particle size of PG-Ana o 3 was generally larger than that of the control untreated Ana o 3 (Figure 6A). However, the difference in particle sizes at the higher PG concentrations did not produce a linear change in particle size. For example, the 3.16 mM PG treatment led to the largest particle size (approximately 1500 nM in diameter), while the 1.0 mM and 10 mM PG treatments produced particles that were closer in average size (approximately 1000 nm). Differences in net Ana o 3 charge were also assessed for each sample, as the loss of positively charged arginine side chains would be expected to alter the charge of the protein. While it was not a linear response, increased PG concentration led to a more negative PG-Ana o 3 average ζ-potential relative to the control (Figure 6B). Negligible change in average ζ-potential was observed at the lowest PG concentration tested (0.1 mM), but at the higher PG concentrations (>0.316–10 mM PG), the average ζ-potential decreased noticeably (Figure 6B). For example, the ζ-potential observed for the 3.16 mM PG sample represents an approximately 26-fold decrease (−42.3 mV) over the average charge observed for Ana o 3 in the absence of PG treatment (∼1.6 mV).

### PG-Ana o 3 Modification Alters Hydrophobic Protein Surface Content

The PG-induced structural perturbations suggested by the antibody binding, CD, and ζ-potential results may be reflected in alterations of the surface properties of PG-Ana o 3. ANS fluorescence increases when it binds to hydrophobic regions on protein surfaces, serving as an indicator of partial protein unfolding or aggregation. ANS emission maxima for both Ana o 3 and PG-Ana o 3 were observed at approximately 520 nm (Figure 7).

**Figure 7:**
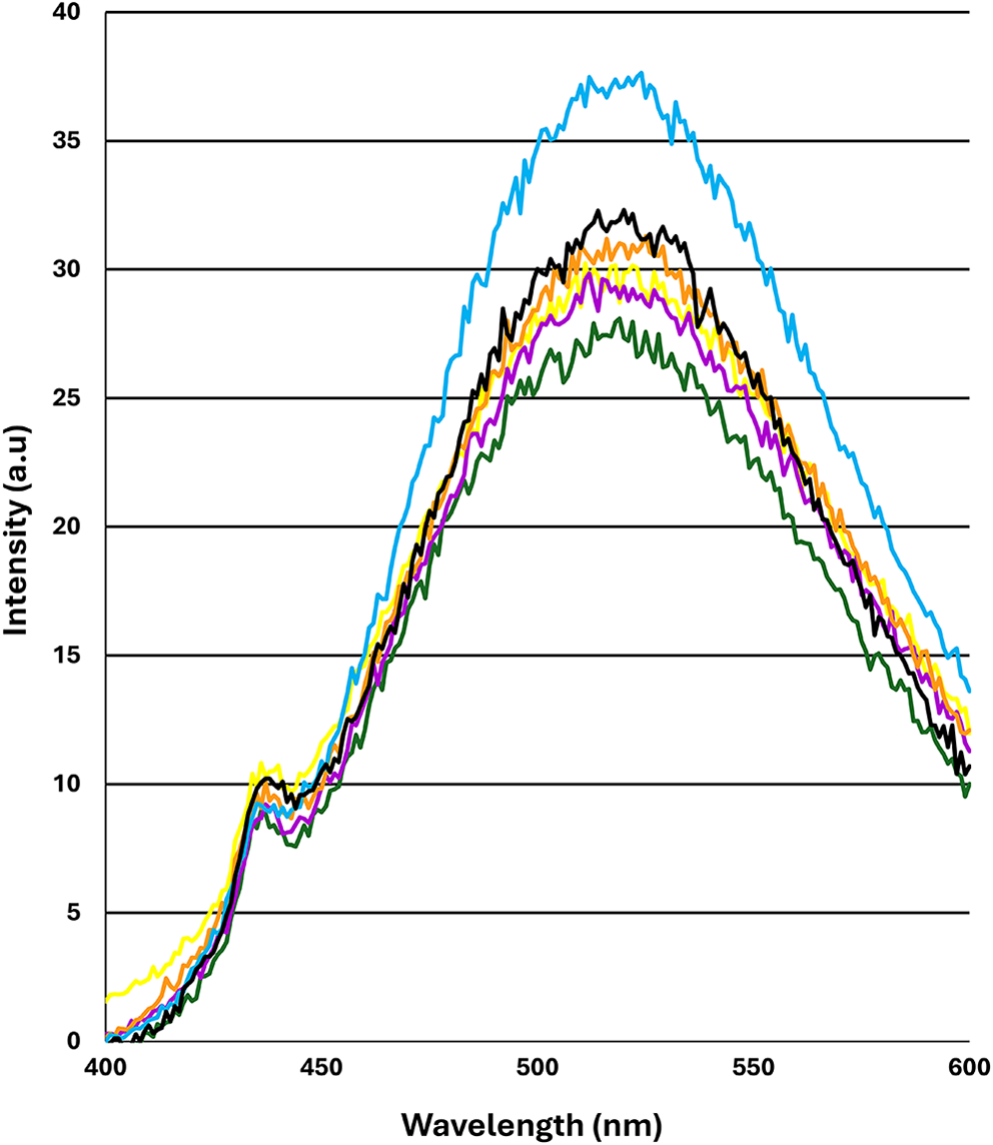
ANS (1-anilino-8-naphthalene sulfonic acid) binding is altered in PG-Ana o 3. Untreated Ana o 3 (yellow) and Ana o 3 samples treated with increasing PG concentrations (0.1 mM-green, 0.316 mM-magenta, 1.0 mM-orange, 3.16 mM-teal, and 10 mM-black) were incubated with ANS to evaluate the hydrophobic surface content of the protein. Samples were excited at 380 nm, and emission was monitored between 400–600 nm with a 5 mm slit width and a +650 mV PMT.

Only relatively minor differences in ANS fluorescence signals were observed between the control and the lowest, 0.1 and 1 mM PG concentrations (Figure 7). However, the fluorescence intensity of PG-Ana o 3 generated with 3.16 mM PG was noticeably increased compared to the other samples. Somewhat surprisingly, the ANS signal then decreased in the 10 mM PG-Ana 3 sample and more closely resembled the untreated control (Figure 7). The ANS binding data suggest that PG treatment only slightly perturbs Ana o 3 conformation relative to the untreated control Ana o 3.

### PG-Ana o 3 Modifications Rigidify Loop Dynamics

To evaluate the effects of PG modification on the Ana o 3 secondary structure and conformational dynamics, molecular dynamics simulations were employed. Based off MS results (Figure 4), Ana o 3 PG-induced modifications at R41, R54, R85, and R111 were modeled (Figure 8A). A wild-type control (WT) was modeled as well as a quadruple-PG to mimic potential PG saturation from MS experiments. PG modeling resulted in increased hydrophobic surface area and overall negative charge (Supporting Table 2).

**Figure 8:**
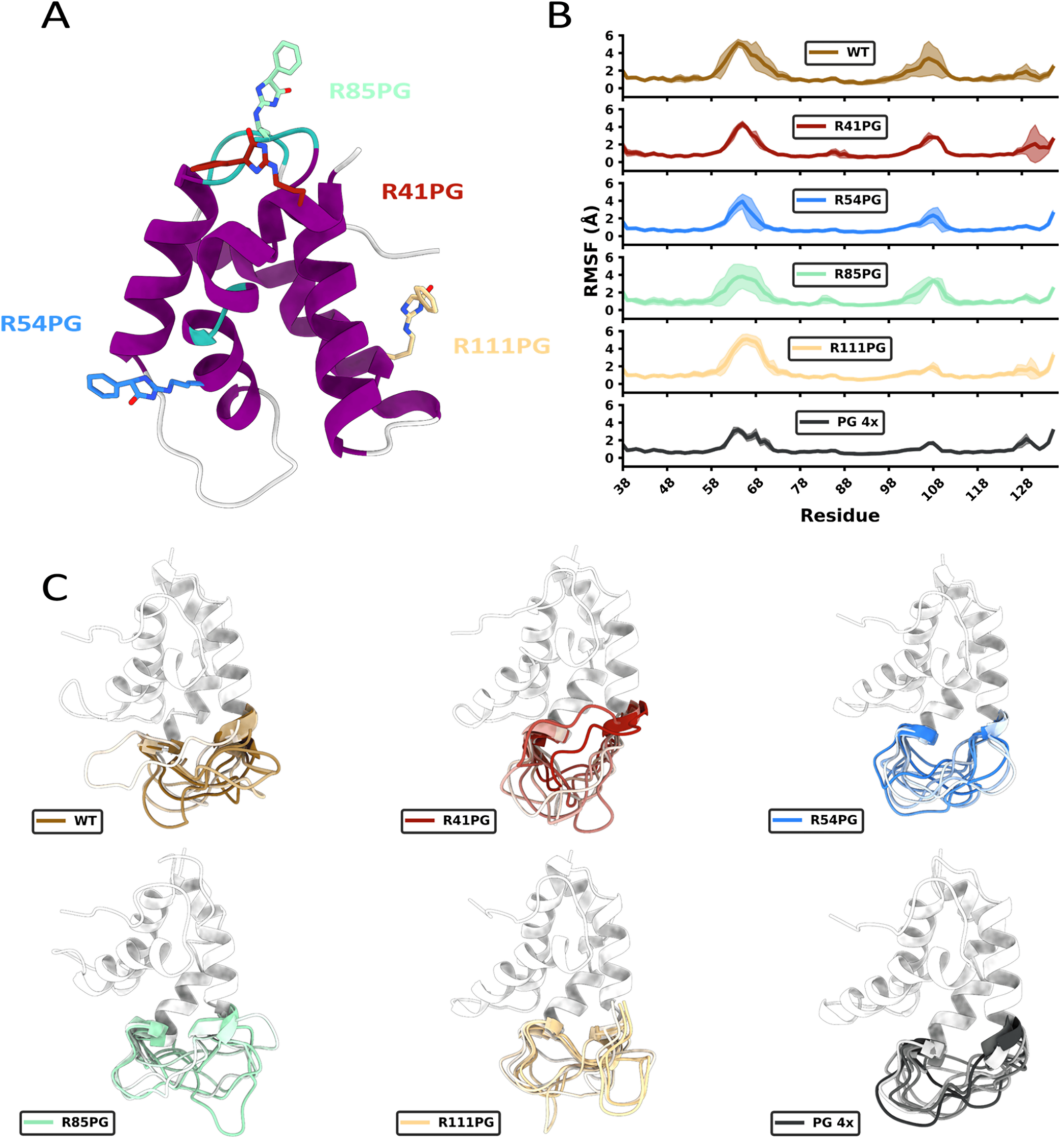
Model of Ana 3 and PG-modified Ana o 3. (A) PG-modified arginine residues R41PG (red), R54PG (blue), R85PG (teal), and R111PG (yellow) arginine residues. (B) Per-residue RMSF values for each modeled variation of PG chemical modification, including quadruple-PG (PG 4×). RMSF data per system is reported as the mean ± standard deviation across three 1000 ns MD trajectories. (C) Representative snapshots of non-native loop fluctuations based off self-organizing map (SOM) clustering of specific non-native loop and hypervariable loop dihedral angles. Gradient-coloring separates loop structures from different SOM clusters, where darker colors represent structures from SOM clusters with greater population.

The Ana o 3 model used the Brazil Nut Ber e 1 structure as a template (RSCB PDB ID: 2LVF).^43^ Like other 2S albumins, the modeled Ana o 3 structure is described by the canonical four-helix fold tethered by four disulfide bonds. Between helices 1 and 2 is the non-native loop (residues E59–N67). NMR experiments have shown the 2S albumin non-native loop to have ps-ns scale backbone dynamics, which are the fastest among the structures.^43^ Similarly, our MD simulations show the non-native loop to have the largest fluctuations along the ns time scale (Figure 8B). Average non-native loop RMSF values for WT (3.74 ± 0.83) and R111PG (3.50 ± 0.68) were the largest, whereas the remaining single PG modification RMSF values ranged from ∼2.5 to 3.0 Å. Quadruple-PG showed the greatest rigidification, with an average RMSF of 2.15 ± 0.30 Å. However, residues Q68 and E75 for WT and R111PG, adjacent to the non-native loop, expressed fluctuations that were ∼0.62 to 2.67 Å greater than the remaining systems.

Between helices 3 and 4 is the hypervariable loop (residues E103–G107). The hypervariable loop has previously been described as the immunodominant epitope of allergenic 2S albumin structures.^72,73^ Average hypervariable loop RMSF for WT was the greatest (2.62 ± 1.26 Å), followed by R85PG (2.17 ± 0.96 Å) and R41PG (2.04 ± 0.33 Å); the remaining systems expressed average RMSFs between ∼1.15 to 1.56 Å. Similar to results for the non-native loop, hypervariable loop-adjacent residues on helices 3 (R97–Q102) and 4 (E108–E112) were more flexible in the WT than for PG systems (except R85PG). Here, the above-mentioned adjacent residues for WT are ∼0.41 to 1.03 Å and ∼0.59 to 1.49 Å more flexible on helices 3 and 4, respectively.

Taken together, MD simulations showed a diverse sampling of non-native loop structures, where the flexibility was reduced by most forms of PG modification (Figure 8C). For single PG modifications, R54PG rigidified non-native loop dynamics the most, whereas R111PG rigidified hypervariable loop dynamics the most. Otherwise, DSSP calculations of MD trajectories showed variability in secondary structure classification, with greater changes occurring along the hypervariable region and helix 4 (Supporting Figures 4–6).

## Discussion

Protein modification in foods due to processing, storage, or spoilage can alter nutritional benefits, sensory qualities, and allergen content. Protein chemistry is frequently used to create perturbations that mimic such modifications for the study of food allergen protein structure and function. In this case, PG was used to selectively modify arginine residues and evaluate several properties of the Ana o 3 cashew nut allergen. The TNBSA data indicate a concentration-dependent reduction in primary amine content in the PG-modified Ana 3 samples, and the MS data presented here support the idea that PG selectively modifies Ana 3 arginine residues. The PAGE migration of the PG-treated Ana o 3 protein was altered compared to the control, but the effect was much more pronounced in the non-DTT-treated samples. The variation in migration between the PG-treated samples suggests that the slowed migration is dependent upon interaction of increased hydrophobic surface patches in the properly folded protein, protein size, protein charge, or possible altered association with the PAGE detergent.

The immunoassay data clearly indicate that PG modification altered anti-Ana o 3 antibody recognition, despite CD, ANS, and modeling that suggest the PG modifications do not appreciably alter Ana o 3 protein structure. The loss of antibody binding among the monoclonal and polyclonal anti-Ana o 3 antibodies assessed here is not surprising, given the sequence/spatial distribution and number of arginine residues modified by PG. Indeed, the 2H5 antibody was made using a peptide (H2N-CQRQFEEQQRFRNCQR–OH) containing the R41, R48, R50, and R54 residues as the immunogen. Loss of 2H5 binding is not surprising because of the choice of peptide sequence used as the immunogen. Similarly, other anti-Ana o 3 monoclonal antibodies developed to characterize Ana o 3 have predicted epitopes that are adjacent to or include arginine residues such as R62 and R65 for the 5B7F8 antibody, and R111 (and possibly R85) for the predicted epitopes of the 6B9C1 and 19C9A2 antibodies.^35^ Furthermore, several Ana o 3 arginine residues lie in or adjacent to published IgE epitopes^11,12^ and would be expected to alter IgE antibody binding. While most antibody binding assays done here indicated decreased binding in the PG-Ana o 3 samples, the signal was elevated in the 5B7F8 blot, but only in the nonreducing agent-treated sample. Conversely, the 5B7F8 signal decreased in the samples containing 5 mM DTT to disrupt the Ana o 3 structure. Though difficult to explain these opposing observations, it is possible that PG treatment could lead to some slight conformational change in Ana 3, allowing greater exposure of the linear 5B7F8 epitope in the sample untreated with DTT. Importantly, the same phenomenon was not observed in the corresponding 5B7F8 ELISA data. The panel of four anti-Ana o 3 monoclonal antibodies, along with the anti-Ana o 3 polyclonal sera, used to characterize PG-modified Ana o 3, serves as a robust surrogate for cashew-allergic IgE, and it is reasonable to expect that IgE from cashew-allergic individuals would also have diminished binding to PG-treated Ana o 3.

Some level of selectivity for PG modification of Ana o 3 arginine residues exists. The R41, R54, R84, and R111 residues reacted with PG at relatively higher intensities compared to other Ana o 3 arginine sites. The R111 residue has previously been shown to be altered via heat-induced carboxyethyl and/or hydroimidazolone modifications.^20^ The relative preference for which specific arginines were observed to be modified is likely based on several factors, including (1) the relative percentage of a given isoform in the purified Ana o 3 preparation used for experiments; (2) the choice of buffer, pH, and other reaction conditions; and (3) the surface position and local availability of specific arginine residues to react with PG.

Overall, the CD findings suggested that PG treatment led to a slight decrease in the regular helical content and a slight increase in disordered regions and certain β-sheet structures. MD simulations with select PG modifications showed slight increases in the disordered regions. ANS is considered a “hydrophobic probe,” and there were relatively minimal changes in ANS fluorescence signal among the samples, indicating some slight change in Ana o 3 surface properties. Of interest was that the 3.16 mM PG treatment caused a noticeable increase in Ana o 3 hydrophobic surface area exposure, which then decreased with the 10 mM PG treatment. All CD and immunoassay experiments were performed with 10 mM PG treatment, reinforcing that PG treatment capable of attenuating antibody interactions poses minimal changes to the Ana o 3 structure. For example, the CD findings suggested that PG treatment led to a slight decrease in the regular helical content and a slight increase in disordered regions and certain B-sheet structures. Further, the MD simulations with selected PG modifications showed only slight increases in disordered regions. Overall, these relatively minor shifts could affect the protein’s stability and compactness. Ultimately, such changes may influence functional properties and molecular interactions similar to what occurs during food processing steps.^74^ The consistent features and subtle changes observed between the control and PG-treated samples underscored the complex balance of structural integrity and flexibility within protein chemistry, highlighting the impact of chemical modifications on the protein secondary structure.

Alterations in Ana o 3 particle size and protein charge were noteworthy. It is straightforward to speculate that the loss of basic arginine side chains through PG modification would result in a more negatively charged protein. Similarly, the PG modification(s) could lead to increased particle size via the increased hydrophobic area and resulting aggregation that may occur due to the association of the hydrophobic surface patches. Further, the added mass and volume due to the PG changes, although relatively small as a percentage of the total protein mass, may also contribute to increased particle size.

While changes in Ana o 3 structure appeared to be relatively minor, PG modification did restrict conformational sampling of the non-native and hypervariable loops compared to unmodified Ana o 3. Given prior reporting of NMR experiments,^43^ PG modification could have greater influence on the hypervariable loop dynamics, but could not be captured within microsecond-long MD simulations. Since the hypervariable loop is responsible for IgE recognition, biased sampling of this structural motif by selective chemical modification could act as a strategy to reduce allergenicity of 2S albumins. However, there is likely nuance to designing modifications. For instance, local PG modification was predicted to rigidify nearby loops the most; however, PG installations had variable effects on the secondary structural preference seen from MD simulations. PG modification of Ana o 3 likely exhibits a combination of intramolecular allosteric and direct antibody binding effects.

Chemically reduced and alkylated 2S albumin proteins have been demonstrated to exhibit unique properties upon modification. For example, modified peanut 2S albumins Ara h 2 and Ara h 6 that have been chemically reduced and alkylated have reduced allergen potency, yet retain their immunogenicity.^24–26^ It has been proposed that these modified peanut allergens could be useful in the development of a new class of immunotherapy reagents.^25,26^ Under the proper reaction conditions, the PG procedure described here could be used to selectively modify specific arginines in Ana o 3 or other food allergens to create molecules with reduced potency. These ‘PG-attenuated’ allergens may also find use as novel therapeutics for allergy treatment. They would be expected to have increased resistance to trypsin digestion and possibly other catabolic enzymes due to PG-induced arginine modifications. Further, while the reduction and alkylation method described by van der Kleij et al.^26^ and Bencharitiwong et al.^25^ results in the loss of Ara h 2 conformation, the PG treatment used here would be expected to have the advantage of preserving Ana o 3 structure. This could be useful as IgE antibodies are often directed toward conformational epitopes.^75–77^

While figures vary, approximately 10–30% of oral immunotherapy patients drop out due to adverse effects, among other reasons.^78–80^ Ana o 3 cognate soluble IgE has emerged as a strong predictor of clinically relevant allergy, but cashew nuts contain three major allergens (including Ana o 1 and Ana o 2), and a majority of patients are sensitive to all three.^81^ Multiallergen IgE profiles are common, and this can complicate desensitization strategies because targeting a single allergen may not fully address the immunologic sensitivities of an individual. However, it is possible that PG-attenuation of Ana o 3 could reduce IgE-binding capacity while preserving T-cell epitopes, allowing for safer immune modulation. Relevant to this, recent findings reveal cross-reactivity between IgE to all three cashew nut allergens (Ana o 1, Ana o 2, and Ana o 3), suggesting that shared structural motifs may drive broader sensitization across nut species.^82^ In this context, a rationally attenuated Ana o 3 could serve as a strategic therapeutic scaffold that could minimize the risk of adverse or anaphylactic responses while also potentially dampening cross-reactive responses through immune tolerance induction. This approach aligns with the growing need for precision allergen immunotherapy that accounts for both intra- and interspecies cross-reactivity, potentially providing a tailored solution for patients with complex sensitization profiles. Following continued study and safety considerations, chemically modified allergens and extracts, such as those described here, may find application as a modified form of immunotherapy. Chemically engineered allergens or foods containing attenuated allergens may serve as steps to reduce exposure sensitivity and minimize adverse reactions for those with extremely severe allergy symptoms.

## Supplementary Material





## Notes

(1) File entitled “SI-PG_Anao3” contains molecular simulation, protein modeling, and mass spectrometry peptide fragmentation data; (2) file entitled “Ana o 3 PG Sup Data File” contains a summary of mass spectrometry peptide and peptide modification observances (PDF)
Ana o 3 PG Sup Data File (XLSX)
