# Supplementary material for: Phenylglyoxal-induced Ana o 3 Modification Reduces Antibody Binding with Minimal Alteration in Protein Structure
Source: J Agric Food Chem. 2025 Sep 17;73(39):25043–56. doi: 10.1021/acs.jafc.5c10372 (PMC12492407; doi:10.1021/acs.jafc.5c10372)
Supplement: Supplementary file 1 [file jf5c10372_si_001.pdf]

Supplementary Information

Phenylglyoxal-induced Ana o 3 Modification Reduces Antibody Binding with Minimal Alteration in Protein Structure

C. Nacaya Brown[1], Tien Thuy Vuong[1], Austin T. Weigle[2,3], Yu-Jou Chou[4], Qinchun Rao[4],

Christopher C. Ebmeier[5], Rebecca A. Dupre[1], Stephen M. Boue[1], Brennan Smith[1], and

Christopher P. Mattison[1]*

[1] Food Processing Sensory Quality, USDA Agricultural Research Service, New Orleans, Louisiana, 70124, USA

[2] Stored Product Insect and Engineering Research Unit, Center for Grain and Animal Health Research, USDA Agricultural Research Service, Manhattan, 66502, USA

[3] SCINet Program and ARS Center of Excellence, Office of National Programs, USDA Agricultural Research Service, Beltsville, MD, 20705, USA

[4] Center for Integrative Nutrition and Food Research, Department of Health, Nutrition, and Food Sciences, Florida State University, 120 Convocation Way, Tallahassee, FL, 32306, USA

[5] Proteomics and Mass Spectrometry Shared Research Resource, Department of Biochemistry, University of Colorado, Boulder, Colorado, 80309 USA

* E-mail: chris.mattison@usda.gov

Table of Contents

Figures and Tables

Table S1 Self-organizing map macrostate cluster populations S-3

Table S2 MOE-predicted protein properties of Ana o 3 and PG-modified Ana o 3 (pH 7.4) S-4

Figure S1 Exploratory data analysis (EDA) of non-native loop dihedral angles S-5

Figure S2 Exploratory data analysis (EDA) of hypervariable loop dihedral angles S-6

Figure S3 Extended mass spectrometry fragmentation analyses of PG-modified sites within Ana o 3 S-7

Figure S4 DSSP secondary structure analyses of alpha-helix (H), 3-10 helix (G), and PPII helix (P) propensities of PG-modified Ana o 3 relative to wild-type control S-10

Figure S5 DSSP secondary structure analyses of bend (S) and H-bond turn (T) propensities of PG-modified Ana o 3 relative to wild-type control S-11

Figure S6 DSSP secondary structure analyses of loop (L) propensities of PG-modified Ana o 3 relative to wild-type control S-12

Supplementary Table 1. Self-organizing map macrostate cluster populations. The top five most populated macrostates are listed along with their the total frequency. Frequency is reported as a percentage of the total number of molecular dynamics frames recorded (N = 30,000). Note that microstate ranking labels are not equivalent to labels assigned during clustering within source data.

Top Macrostates	WT	R41PG	R54PG	R85PG	R111PG	PG 4x
1	25.38	37.14	27.48	20.68	20.60	65.48
2	25.30	10.45	26.92	18.90	11.29	21.06
3	25.15	10.29	13.65	13.65	9.36	7.08
4	9.93	9.97	12.13	5.93	8.17	4.63
5	5.26	8.69	6.65	5.10	6.69	0.88

Supplementary Table 2. MOE-predicted protein properties of Ana o 3 and PG-modified Ana o 3 (pH 7.4)

Predicted Protein Properties of Ana o 3 and PG-modified Ana o 3 (pH 7.4)						
**Description**	**Ana o 3**	**R41PG**	**R54PG**	**R85PG**	**R111PG**	**R41/54/85/111PG**
**Area of hydrophobic protein patch(es)**	70	130	120	70	150	270
**Area of positive protein patch(es)**	150	150	140	100	150	100
**Area of negative protein patch(es)**	160	210	120	160	150	150
**Area of ionic protein patch(es)**	310	360	260	260	300	250
**Protein Mass in kDa**	11.9289	12.045	12.045	12.045	12.045	12.3934
**Structure-based pI Prediction**	8.56	6.81	6.6	6.58	6.67	4.75
**Extinction coefficient at 280 nm**	4970	4970	4970	4970	4970	4970
**Radius of Gyration**	12.81	12.92	12.86	12.83	12.97	13.07
**Hydrodynamic Radius**	17.3	17.51	17.41	17.24	17.59	17.78
**Hydrophobic Surface Area**	2713.7	2921.8	2828.9	2766.4	2915.2	3305.8
**Hydrophilic Surface Area**	2765.2	2713.1	2718.2	2658.2	2798.3	2568.4
**Protein Volume**	10679.9	10793.8	10789	10781.6	10799.5	11097
**Protein Helix Ratio**	57.1	58.2	58.2	58.2	57.1	58.2
**Protein Net Charge**	1.77	-0.09	-0.28	-0.41	-0.25	-4.95
**Zeta Potential at Debye Length**	7.37	0.13	-0.54	-1.13	-0.62	-18.06
**Sequence-based pI Prediction**	6.04	5.63	5.63	5.63	5.63	4.77

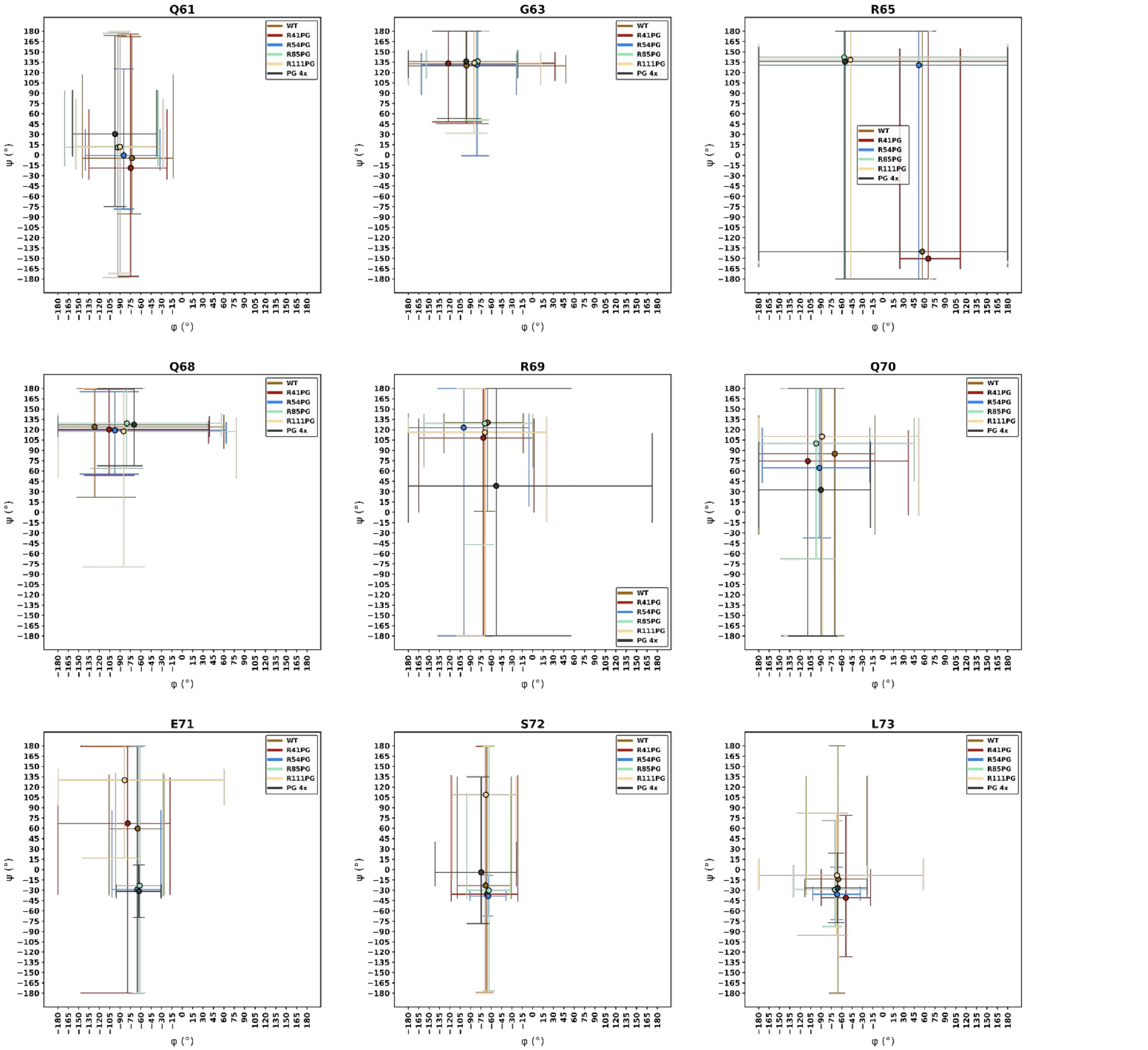

Supplementary Figure 1. Exploratory data analysis (EDA) of non-native loop dihedral angles. From left to right: *top row* (Q61, G63, R65), *middle row* (Q68, R69, Q70), *bottom row* (E71, S72, L73). Distribution differences were considered functionally significantly different if the means were at least 30° different from another distribution in either the phi (ϕ) or psi (ψ) dimensions. Backbone dihedral calculations were pooled across each trajectory replica per system, totaling to 30,000 frames as individual data points.

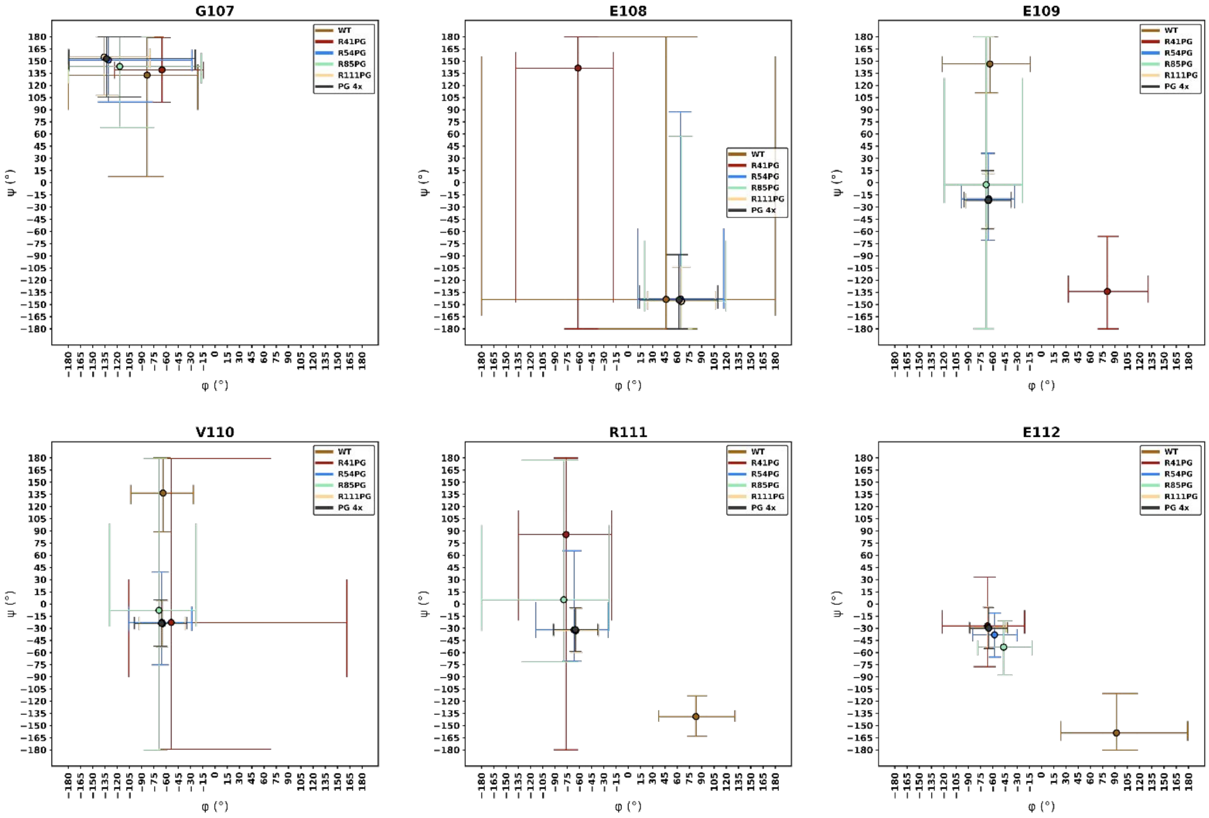

Supplementary Figure 2. Exploratory data analysis (EDA) of hypervariable loop dihedral angles. From left to right: *top row* (G107, E108, E109), *bottom row* (V110, R111, E112). Distribution differences were considered functionally significantly different if the means were at least 30° different from another distribution in either the phi (ϕ) or psi (ψ) dimensions. Backbone dihedral calculations were pooled across each trajectory replica per system, totaling to 30,000 frames as individual data points.

MS/MS Fragmentation of **EQSCQRQFEEQQR** Ana o 3 peptide.
Underlined arginine 41 (R41) modification site is supported by fragmentation data.

Selected scored matches

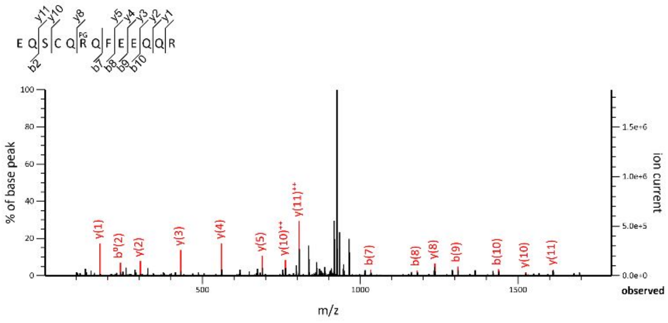

All matches

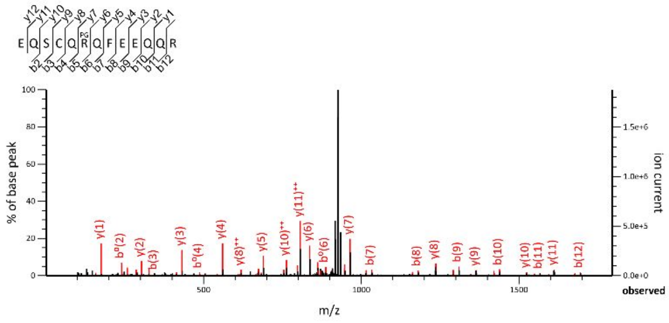

Score	Mr(calc)	Delta	Sequence	Site Analysis
78.3	1867.7907	0.0051	EQSCQRQFEEQQR	PG R6 100.00%
6.6	1867.7907	0.0051	EQSCQRQFEEQQR	PG R13 0.00%

MS/MS Fragmentation of **NCQRYVK** Ana o 3 peptide.
Underlined arginine 54 (R54) modification site is supported by fragmentation data.

Selected scored matches

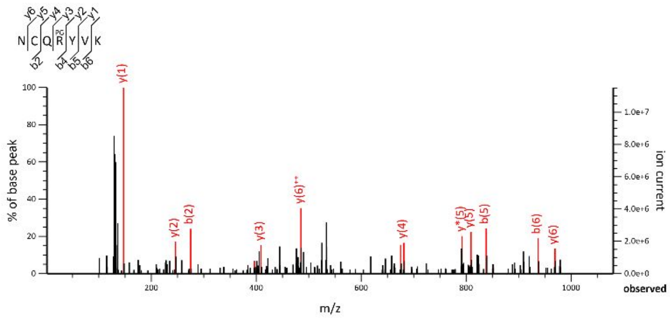

All matches

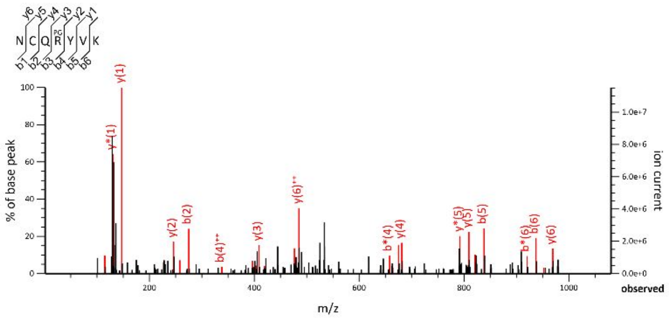

Score	Mr(calc)	Delta	Sequence
47.7	1082.4967	0.0024	NCQRYVK

MS/MS Fragmentation of **ECCQELQEVDRR** Ana o 3 peptide.
Underlined arginine 85 (R85) modification site is supported by fragmentation data.

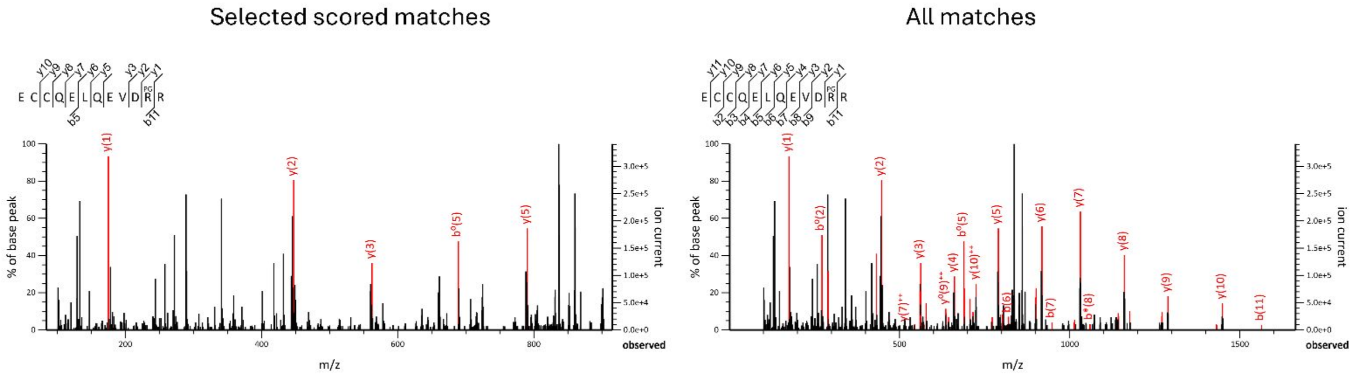

Score	Mr(calc)	Delta	Sequence	Site Analysis
74.4	1736.7246	-0.0018	ECCQELQEVDRR	PG R11 99.60%
50.5	1736.7246	-0.0018	ECCQELQEVDRR	PG R12 0.40%

MS/MS Fragmentation of **GEEVRELYETASELPR** Ana o 3 peptide.
Underlined arginine 111 (R111) modification site is supported by fragmentation data.

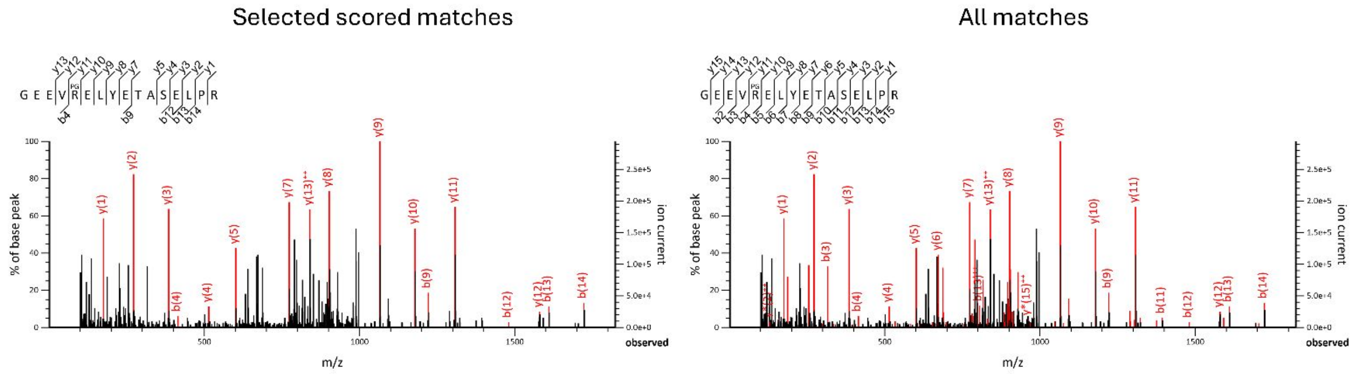

Score	Mr(calc)	Delta	Sequence	Site Analysis
116.3	1992.9428	0.0083	GEEVRELYETASELPR	PG R5 100.00%

Supplementary Figure 3. Extended mass spectrometry fragmentation analyses of selected PG-modified sites within Ana o 3. Mascot ions with scores above identity threshold requirements are shown, confirming the indicated peptide sequence identities. MS/MS

fragmentation analyses indicate specific underlined arginine residues were modified by PG. This includes, from top to bottom, R41PG, R54PG, R85PG, and R111PG modification sites.

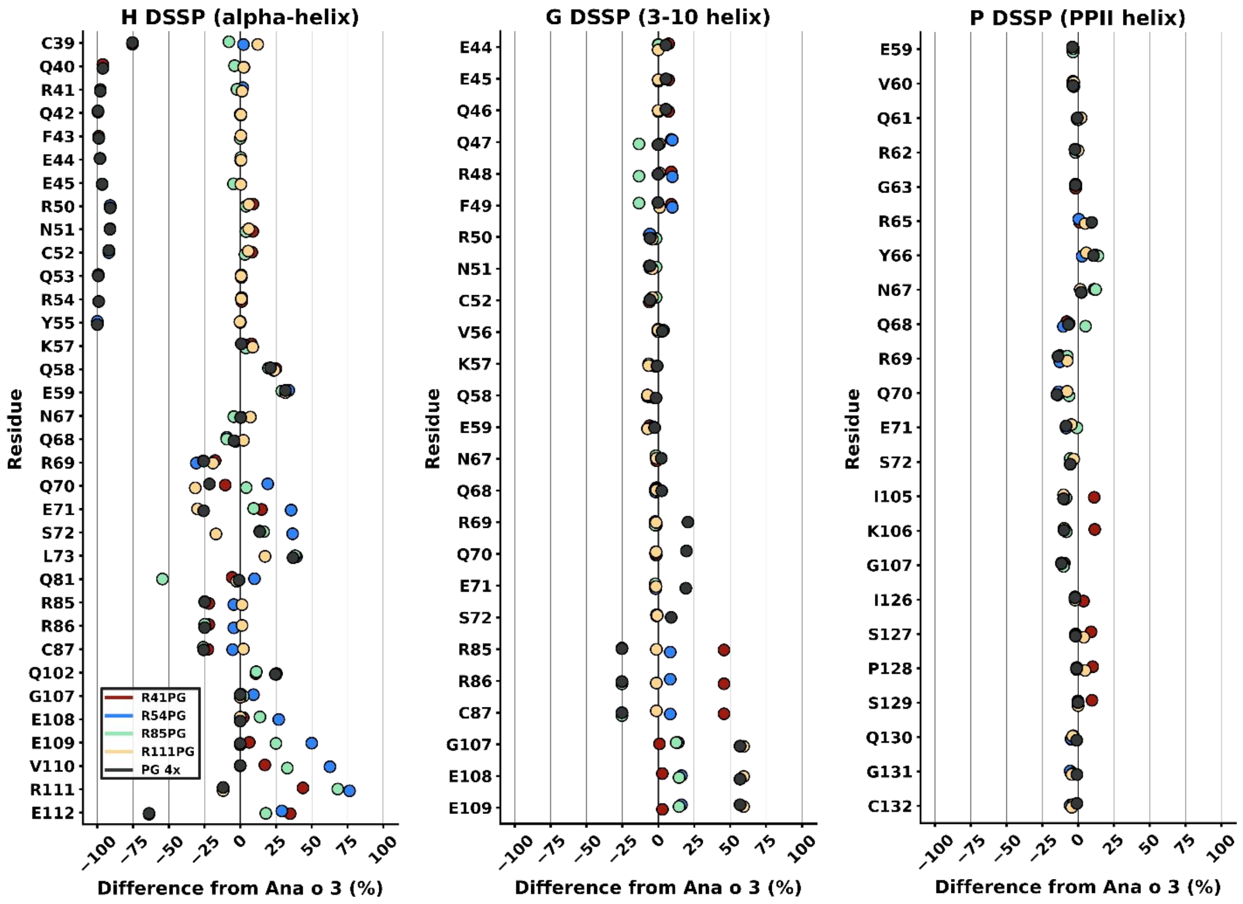

Supplementary Figure 4. DSSP secondary structure analyses of alpha-helix (H), 3-10 helix (G), and PPII helix (P) propensities of PG-modified Ana o 3 relative to wild-type control. Raw DSSP values were calculated as the percentage observed within each molecular dynamics trajectory, and then averaged across the triplicate trajectories per system. Triplicate-averaged values were then subtracted against the wild-type control (unmodified) values as background. Select residues are shown to emphasize differences, where the changing residues vary based off DSSP classification.

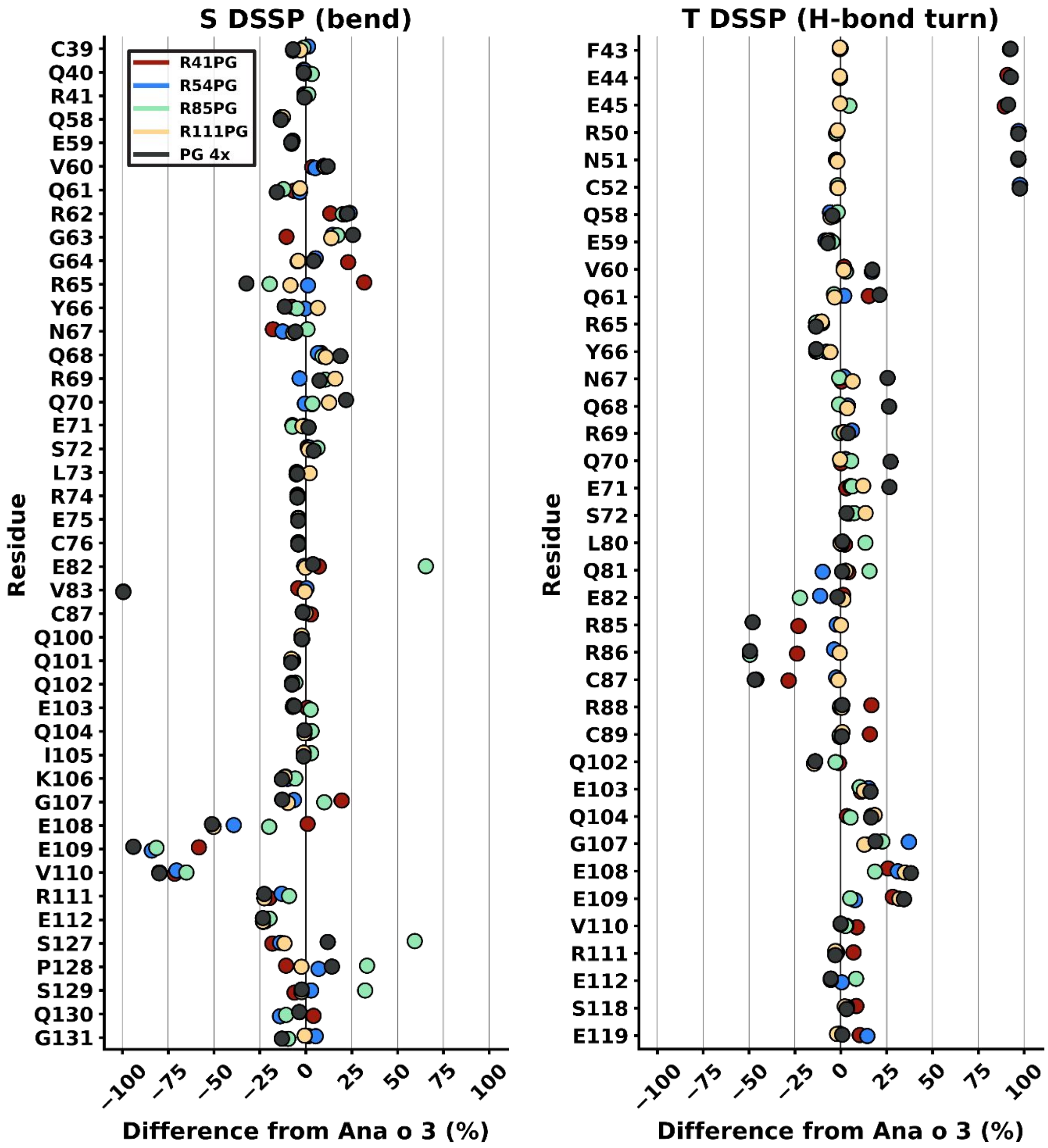

Supplementary Figure 5. DSSP secondary structure analyses of bend (S) and H-bond turn (T) propensities of PG-modified Ana o 3 relative to wild-type control. Raw DSSP values were calculated as the percentage observed within each molecular dynamics trajectory, and then averaged across the triplicate trajectories per system. Triplicate-averaged values were then subtracted against the wild-type control (unmodified) values as background. Select residues are shown to emphasize differences, where the changing residues vary based off DSSP classification.

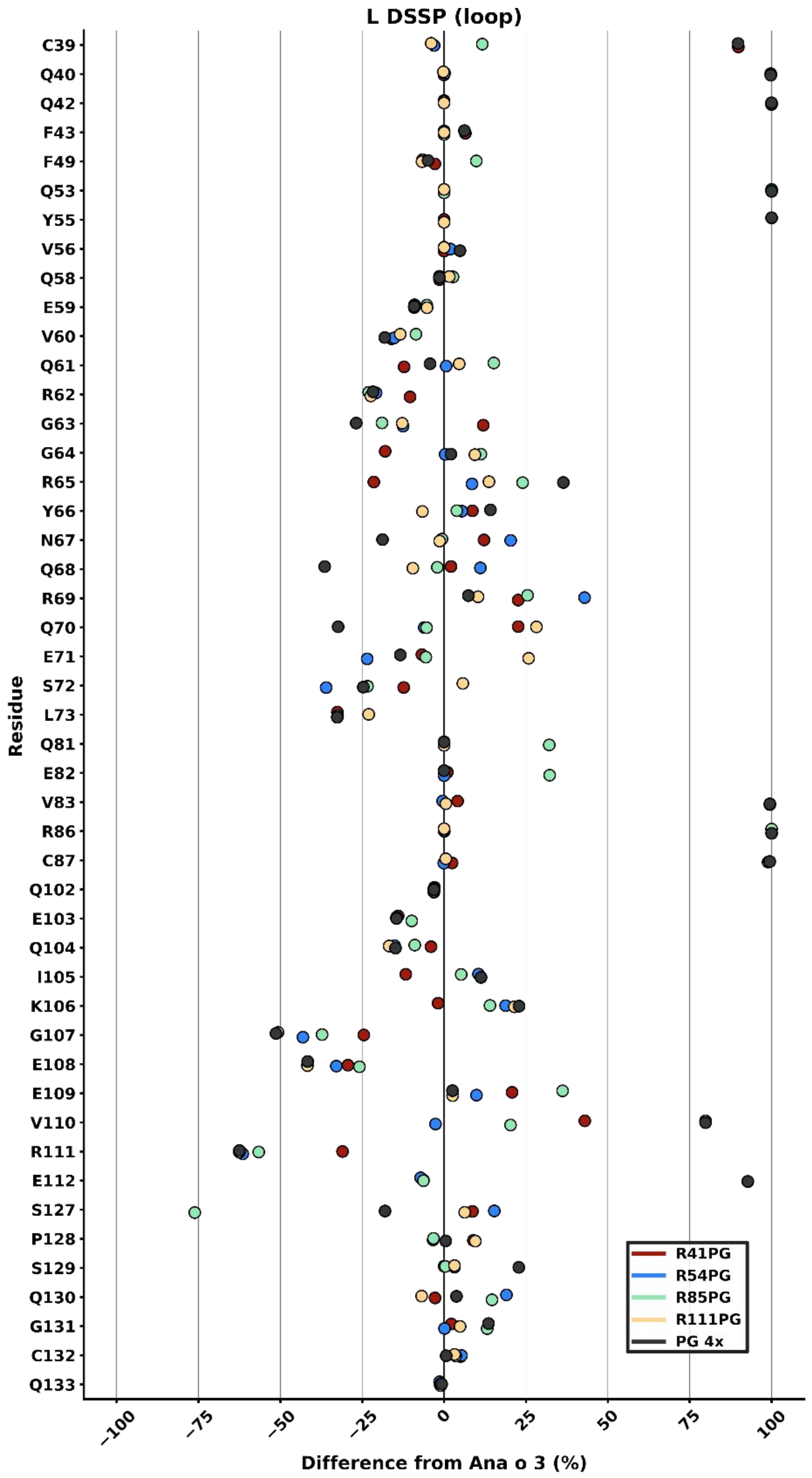

Supplementary Figure 6. DSSP secondary structure analyses of loop (L) propensities of PG-modified Ana o 3 relative to wild-type control. Raw DSSP values were calculated as the percentage observed within each molecular dynamics trajectory, and then averaged across the triplicate trajectories per system. Triplicate-averaged values were then subtracted against the wild-type control (unmodified) values as background. Select residues are shown to emphasize differences, where the changing residues vary based off DSSP classification.
